# Protective effect of Shenqi Wenfei Formula against lipopolysaccharide/cigarette smoke-induced COPD in Rat based on gut microbiota and network pharmacology analysis

**DOI:** 10.3389/fmicb.2024.1441015

**Published:** 2024-11-19

**Authors:** Mengyao Shi, Qian Xue, Jinghui Xie, Qinjun Yang, Jiabing Tong, Jie Zhu, Yating Gao, Xiao Ma, Di Wu, Zegeng Li

**Affiliations:** ^1^Anhui University of Chinese Medicine, Hefei, China; ^2^Chinese Medicine Respiratory Disease Prevention Institute, Hefei, China; ^3^Anhui Province Key Laboratory of the Application and Transformation of Traditional Chinese Medicine in the Prevention and Treatment of Major Pulmonary Diseases, Hefei, China; ^4^First Affiliated Hospital of Anhui University of Chinese Medicine, Hefei, China

**Keywords:** COPD, SQWF, network pharmacology, 16S rRNA sequencing, *Parabacteroides*, NETs

## Abstract

**Introduction:**

The incidence of chronic obstructive pulmonary disease (COPD) appears to be increasing and evidence suggests that the intestinal flora may play a causative role in its development. Previous studies found that the Shenqi Wenfei Formula (SQWF) can regulate pyroptosis via the NLRP3/GSDMD pathway, thereby reducing the inflammatory response in the lungs of COPD model rats. However, there is no information on whether the drug's effects are associated with intestinal flora. Therefore, this study investigates whether the effects of SQWF are mediated through the regulation of intestinal flora, aiming to elucidate the underlying mechanisms of its therapeutic impact on COPD.

**Methods:**

COPD was induced in rats using lipopolysaccharide and cigarette smoke, followed by intragastric administration of SQWF or physiological saline The targets of SQWF, associated signaling pathways, and key bacterial groups were investigated using 16S rRNA sequencing, network pharmacology, and bioinformatics techniques. The prediction results were validated using quantitative reverse transcription PCR, western blotting, and immunofluorescence, among other methods.

**Results:**

SQWF treatment was found to alleviate COPD in model rats. Treatment was also observed to restore the balance of the intestinal flora in the rats, especially by reducing the abundance of *g_Parabacteroides*. Bioinformatics predictions identified *g_Parabacteroides* metabolites, RelA, HDAC1, and enriched neutrophil extracellular trap formation pathways as core targets of SQWF in COPD. qRT-PCR and Western blotting results showed that SQWF treatment reduced ReLA and HDAC1 mRNA and protein expression, along with decreased myeloperoxidase and neutrophil elastase levels in the nucleus.

**Conclusion:**

Treatment with SQWF was found to restore the imbalance of intestinal *g_Parabacteroides* in COPD and also regulate the expression of the ReLA and HDAC1 genes, thereby reducing pulmonary neutrophil extracellular traps and alleviating lung inflammation.

## 1 Introduction

Chronic obstructive pulmonary disease (COPD) is characterized by chronic inflammation and airway obstruction, leading to progressive and irreversible impairment of lung function (Wang L. et al., 2023). The global prevalence and mortality of COPD remain high, causing significant burdens on both individuals and society (Li et al., 2020). Chronic smoking (CS) significantly contributes to the development of COPD by inducing the release of endogenous cytokines and risk-related pattern factors. This directly damages airway epithelial cells, activates non-specific inflammatory responses, and leads to further pathological changes in the lungs (Decramer et al., 2012). Further, COPD patients show abnormal pulmonary gas exchange functions, causing hypoxia in intestinal epithelial cells, as well as increased permeability and microbial disorders (Fricker et al., 2018).

A recent study has shown that intestinal microorganisms contribute significantly to COPD onset (Lai et al., 2022). Although the lungs and intestines are not anatomically identical, both contain rich bacterial communities. The composition of the intestinal microflora affects the immune response and changes in the relative abundance of microorganisms in both the intestine and the lungs can lead to dysbiosis and adversely affect the body's immunity (Budden et al., 2017). A study in mice has shown that transplantation of fecal microflora from patients with COPD to healthy mice can up-regulate the expression of inflammatory factors and aggravate lung inflammation (Li N. et al., 2021). A clinical study has shown that changes in the intestinal microflora represent a risk factor for COPD, independent of conventional risk factors (Liu et al., 2023). The intestinal microflora and its metabolites can regulate inflammation and immune function, with its lipid metabolites affecting host physiology (Xie et al., 2022). Therefore, the restoration of the intestinal flora and normalizing its metabolite levels may be an effective strategy for treating COPD (Hu et al., 2020; Qu et al., 2022).

Traditional Chinese medicine can improve lung function in patients with COPD by modulating the expression of inflammatory factors in serum and alleviating symptoms, serving as an important adjunct to conventional clinical treatments for COPD (Li W. et al., 2010; Shen et al., 2019; Li et al., 2021; Chen et al., 2022). Shenqi Wenfei Formula (SQWF) is used as a complementary therapy for patients with COPD and is effective. SQWF can improve the pulmonary inflammatory response in COPD model rats by regulating the NLRP3/GSDMD pathway and inhibiting the release of inflammatory factors (Wu D. et al., 2023). However, the effects of SQWF treatment on the intestinal microecology in COPD are unknown.

**Figure 1:**
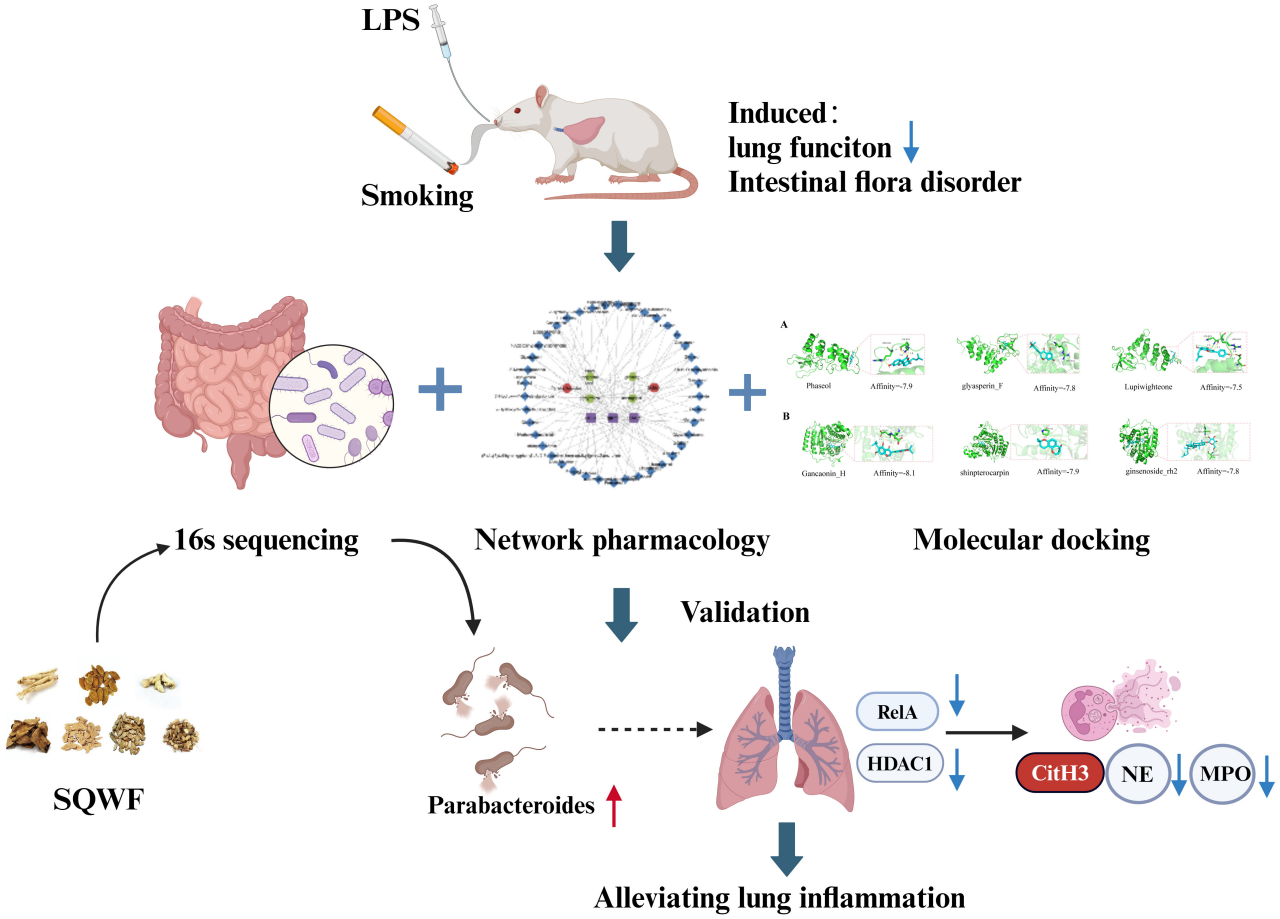
The combination of CS and LPS can adversely affect both lung function and the balance of the intestinal flora in rats. SQWF can improve lung function by regulating the intestinal flora. This mechanism may be associated with increased abundance of *Parabacteroides*, resulting in the indirect regulation of ReLA and HDAC1 and reducing NET formation.

This study hypothesized that SQWF can treat COPD by restoring the balance of the intestinal flora. Using 16S rDNA sequencing, network pharmacology, and bioinformatics, it was found that regulation of the intestinal microflora was key to the effects of SQWF in the treatment of COPD, thus providing a new approach for treating the disease. We put on the flow chart of the research process is displayed in Figure 1.

## 2 Materials and methods

### 2.1 Materials

#### 2.1.1 Animals

Thirty SPF-grade male Sprague-Dawley rats weighing 180 ± 20 g were purchased from Dongfang Breeding Co., Ltd. [Pizhou City, China; License No.: SCXK(Su)2017-0003]. The animals were housed in the Laboratory Animal Center of the Anhui University of Chinese Medicine (constant temperature of 25°C, 12-h light/dark cycle). The study was approved by the Animal Ethics Committee of the Anhui University of Chinese Medicine (AHUCM-rats2021087).

#### 2.1.2 Drug composition and preparation

SQWF were obtained from the First Affiliated Hospital of Anhui University of Traditional Chinese Medicine and the composition is shown in Table 1.

**Table 1:** Main components of the SQWF.

**Chinese name**	**Latin name**	**Family**	**Dosage/g**
Ren Sheng	Panax Ginseng C. A. Mey	Araliaceae	10
Huang Qi	Hedysarum multijugum Maxim	Fabaceae	15
Gan Jiang	Zingiberis Rhizoma	Ginger	6
Chen Pi	Citri Reticulatae Pericarpium	Rutaceae	10
Bai Zhu	Atractylodes Macrocepha la Koidz	Compositae	10
Jie geng	Platycodon Grandiforus	Campanulaceae	6
Gan Cao	Licorice	Leguminosae	6

For preparation, the SQWF decoction was prepared into a medicinal solution with a crude drug concentration of 0.567 g·mL^−1^ (1 mL·100 g^−1^). In a previous studiy, 0.567 g·mL^−1^ (equivalent to 1 mL per 100 g) was identified as the medium-dose group, demonstrating significant improvements in inflammation-related indicators and lung function in COPD rats, and some inflammatory indicators showed no significant difference compared to the high-dose group (Wu D. et al., 2023). This formulation was then stored at 4°C.

### 2.2 Experimental grouping and model preparation

The rats were randomly divided into three groups, namely, the control (Con), model (Mod), and SQWF groups, with 10 rats in each group. The COPD model was established in the Mod and SQWF groups by exposing rats to whole-body cigarette smoke (CS) in combination with lipopolysaccharide (LPS) administration via a tracheal drip method. LPS (200 μL, 1 mg·kg^−1^) was injected into the airway on the 1st and 14th days of modeling. The rats were placed in a homemade fumigation box for 1 h per day from the 2nd to the 28th day of the modeling period (except for the 14th day), and the Con group was left untreated. On the 29th day of modeling, SQWF (5.67 g·kg^−1^) was administered by gavage to rats in the SQWF group, while rats in the Con and Mod groups received an equal volume of saline. Each group was gavaged once a day for 14 days.

### 2.3 Lung function test

The rats were fasted for 24 h following the last treatment and were then injected intraperitoneally with 0.3% pentobarbital sodium (1 mL/kg). An animal lung function analysis system (AniRes2005, Bellanbo Technology Co., LTD,Beijing) was used to measure lung function in rats. After tracheotomy and double-pass intubation, the rats were placed in a supine position in a closed volumetric tracing box to ensure that the tracheal intubation and the airway of the tracing box were connected smoothly. Following stabilization of the airway pressure waveform, the forced expiratory volume in 0.3 s (FEV0.3), forced vital capacity (FVC), and peak expiratory flow (PEF) were measured.

### 2.4 H&E

Lung tissue samples were subjected to fixation, followed by dehydration with alcohol and xylene. The samples were then embedded in paraffin, and sectioned into 4 μm thick slices using a paraffin microtome.

The sections were stained with hematoxylin & eosin (H-E) for histopathological evaluation. Measurements of alveolar enlargement were carried out using H&E-stained slides. Microtome sections from H&E-stained sections of paraffin–embedded mouse lungs were digitally imaged.

### 2.5 16S rRNA gene sequencing

#### 2.5.1 Sample collection

Gut contents (GC) were collected from each rat using sterile instruments. The fresh samples were placed in cryotubes and rapidly frozen in liquid nitrogen, followed by storage at −80°C for further microbiological analysis.

#### 2.5.2 DNA extraction and PCR amplification

The sequencing and data processing were performed by Shanghai Applied Protein Technology Co. Ltd.

DNA was extracted from the GC samples and the concentrations were measured. Specific primers were used for PCR amplification of selected V3-V4 variable regions while using Barcode and high-fidelity DNA polymerase.

#### 2.5.3 Library construction and sequencing

The amplification products were electrophoresed and the target fragments were recovered (Quant-iT Pico Green dsDNA Assay Kit). The PCR-amplified products and those recovered from preliminary electrophoresis were assessed and quantified using a microplate reader (-FLx800, BioTek, Winooski, WI, USA). The quantities of each sample used for sequencing were then mixed in proportions according to the sequencing requirements. The libraries were constructed while using The TruSeq Nano DNA LT Library Prep Kit (Illumina, San Diego, CA, USA). Quality assessment of the constructed libraries was conducted using Agilent Bioanalyzer 2100 (Santa Clara, CA, USA) and Promega QuantiFluor (Madison, WI, USA), followed by sequencing.

#### 2.5.4 Bioinformatics analysis

Raw data were obtained in FASTQ format (Reyon et al., 2012). Following the download of the data, the raw sequence reads were analyzed using Cutadapt software to remove the primer sequences. The quality-checked paired-end raw data were then analyzed using DADA2 with the default parameters of QIIME 2, resulting in the generation of representative sequences and abundance data (Rognes et al., 2016). The RDP Classifier software was utilized to compare the data against the Silva database (version 138), employing a confidence threshold of >0.7 (Wang Q. et al., 2007).

QIIME2 software was used to analyze the alpha and beta diversity in the samples. The alpha diversity of the samples was assessed using the Chao1 and Simpson indices (Chao and Bunge, 2002; Hill et al., 2003). Principal coordinate analysis (PCoA) plots were employed to assess beta diversity using Bray-Curtis analysis, as well as Unweighted and Weighted UniFrac distances. A distance matrix calculated in R was used to assess sample diversity. Variance analysis was performed using the Kruskal-Wallis statistical algorithm based on the R package. Species abundance spectra were analyzed for variance using LEfSe.

### 2.6 Network pharmacology analysis

The terms “Panax Ginseng C. A. Mey,” “Hedysarum Multijugum Maxim,” “Zingiberis Rhizoma,” “Citrus Reticulata,” “Atractylodes Macrocephala Koidz,” “Platycodon Grandiforus,” and “licorice” were entered into the Traditional Chinese Medicine Systems Pharmacology (https://www.tcmsp-e.com/#/database, TCMSP) as keywords. The filters with oral bioavailability ≥ 30% and drug-like ≥ 0.18 were used to identify the active ingredients (Ru et al., 2014). The corresponding SMILES terms were then searched in the PubChem database (https://pubchem.ncbi.nlm.nih.gov/), and the targets of the relevant components were predicted using the Swiss Target Prediction database (http://www.swisstargetprediction.ch/) (Wang et al., 2017).

The identification of COPD targets was performed usingGeneCards (https://www.genecards.org/), OMIM (https://www.omim.org/), and DisGeNET (https://www.disgenet.org/search) (Amberger et al., 2015; Stelzer et al., 2016; Pinero et al., 2017). The keyword “chronic obstructive pulmonary disease” was used, with the median score as the critical point, and duplicate targets were excluded from the subsequent analysis.

LEfSe analysis was carried out to identify differentially abundant bacteria between the groups, using LDA values >2 to detect bacterial genera with significant differences, as well as those showing reduced abundance in the model group compared to the normal and SQWF groups. The names of these bacteria were submitted to gutMGene (http://bio-computing.hrbmu.edu.cn/gutmgene/search.dhtml) to identify metabolites associated with the genera (Cheng et al., 2022). The corresponding SMILES name was identified using the PubChem database and the Swiss Target Prediction (STP) database was used to predict the targets of the metabolites. The online VENN tool (http://bioinformatics.psb.ugent.be/webtools/Venn/) was then used to identify overlaps between the metabolite targets and the predicted COPD targets, followed by the construction of a Venn diagram. The targets identified by the Venn diagram were then imported into the STRING database (https://cn.string-db.org/), the network compiled using cytoHubba, and the key points analyzed using Degree Product (DP), EcCentricity (EC), and Radiality (RA) topology analysis methods Gene (hub gene) and target network (Szklarczyk et al., 2017). A protein-protein interaction (PPI) network was constructed using Cytoscape 3.10.1, showing the associations among the disease, bacterial metabolites, pathways, active components, and hub genes. The intersection targets were entered into the Metascape database for Gene Ontology (GO) and Kyoto Encyclopedia of Genes and Genomes (KEGG) enrichment analyses.

### 2.7 Molecular docking

Obtain the crystal structures of ReLA and HDAC1 proteins from the PDB database (https://www.rcsb.org/). PyMOL-2.1.0 software was employed to optimize the target proteins by removing water molecules and small molecule ligands. AutoDock Tools-1.5.6 was then used to perform hydrogenation and charge addition, with the processed structures saved in pdbqt format. Based on previous network pharmacology results, compounds in SQWF were screened for their relation to the hub genes (ReLA, HDAC1). Finally, the vina-2.0 module within PyRx software was utilized to perform molecular docking between the proteins and the compounds, calculating the binding energy and generating output files. PyMOL software was used for visualizing the results. The Affinity (kcal/mol) value represented the binding strength between the two; lower binding energy indicated more stable ligand-receptor binding (lower binding energy indicates better binding).

### 2.8 Ultrastructural observations by transmission electron microscopy

The upper parts of the right lungs were washed with normal saline. After absorption of excess fluid with filter paper, a scalpel was used to cut a tissue block no larger than 1 mm^3^. The block was placed immediately in electron microscopy fixative for 4 h, after which it was rinsed three times with phosphate-buffered saline (PBS), followed by the addition of 1% osmic acid. After discarding the osmic acid, the sample was rinsed three times with PBS, dehydrated in an ethanol gradient, and permeated with 1:1 propylene oxide:epoxy resin at room temperature for 2 h. The sample was then permeated with pure epoxy resin 812 at room temperature for 2 h, embedded, and sectioned into 70-nm ultra-thin sections, followed by staining with lead citrate for 30 min. The stained sections were evaluated and imaged under transmission electron microscopy (TEM).

### 2.9 Real-time fluorescence quantitative PCR

Total RNA was extracted from the samples using a Rapid RNA extraction kit (AC0202, Sparkjade^®^ Co, Shandong), and the concentration and purity of the RNA were assessed using a UV-visible spectrophotometer. The RNA was reverse-transcribed to cDNA using an RNA reverse-transcription kit, and the cDNA was amplified by PCR. The reaction steps were as follows: pre-denaturation at 95°C, 10 min; denaturation at 95°C, 15 s; annealing at 58°C, 30 s; extension at 72°C, 30 s; cycle 40 times. The primer sequences were: ReLA, Forward TCTACAGGCAGAAGGCGGAGGA, Reverse TGGCGCTTGACACCACAGGTTC, 185 bp; *HDAC1* Forward CAGAAAAACTTACACTGCCC, Reverse GAAACGCGCTGTTTTATTAC, 119 bp; β*-actin* internal reference, Forward CCCATCTATGAGGGTTACGC, Reverse TTTAATGTCACGCACGATTTC, 150 bp. The relative expression of the mRNA was calculated using the 2^−ΔΔCt^ method.

### 2.10 Western blotting

The rat lung tissues were stored at −80°C until analysis. The tissues were lysed, ground in a freezing grinder, and centrifuged at 12,000 rpm for 15 min at 4°C. The protein concentrations of the supernatant were quantified using the BCA method. Appropriate volumes of protein were mixed with 5 × loading buffer and subjected to separation on 8% SDS-PAGE gels, followed by transfer to PVDF membranes at 300 mA for 2 h. The membranes were blocked with 5% skim milk for 1 h, and incubated with primary antibodies against neutrophil elastase (NE) (1:1,000, Abcam, Cambridge, UK, ab310335), CitH3 (1:1,000, Abcam, ab281584), myeloperoxidase (MPO) (1:4,000, Proteintech, China, 66177-1-Ig), NF-κB p65 (1:2,000, ImmunoWay Biotechnology, Plano, TX, USA, YM3111), NF-κB p-p65 (1:1,000, ImmunoWay Biotechnology, YP0191), HDAC1 (1:1,000, Abcam, ab280198), and the internal reference GAPDH (1:2,000, Zsbio, Beijing, China, TA-08) overnight at 4°C. After three washes with PBST, the membranes were incubated with the secondary antibody for 1 h at room temperature, followed by three further washes with PBST. Band visualization was carried out using an ECL luminescence kit and band intensities were quantified using ImageJ software. The results were expressed relative to the expression of the internal control, GAPDH.

### 2.11 Immunofluorescence

The freshly isolated lung tissue was fixed in 4% paraformaldehyde for 24 h, before dehydration in an ethanol gradient, clearing with xylene, and embedding in paraffin. The samples were sliced into 5 μm sections using a microtome, followed by placing them in warm water and floating onto anti-detachment slides. The sections were then baked at 60°C for at least 2 h, followed by dewaxing, antigen retrieval, and sealing with serum. Press 1: Anti-NE and anti-MPO antibodies (1:1,000 dilution) were then added dropwise and incubated overnight at 4°C, followed by dropwise addition of the secondary antibody (1:2,000 dilution) and incubation at room temperature for 1 h. The nuclei were counterstained with DAPI (1:7,000) for 15 min, after which the slide was covered evaluated, and imaged using a fluorescence microscope.

### 2.12 Statistical analysis

Measurement data were expressed as mean ± standard deviation and were compared using a one-way analysis of variance. SPSS software was used for data analysis while GraphPad Prism was used for visualization. *P*-values < 0.05 were considered statistically significant.

## 3 Results

### 3.1 SQWF improves lung function and alleviates pathological damage in lung tissue from COPD rats

**Figure 2:**
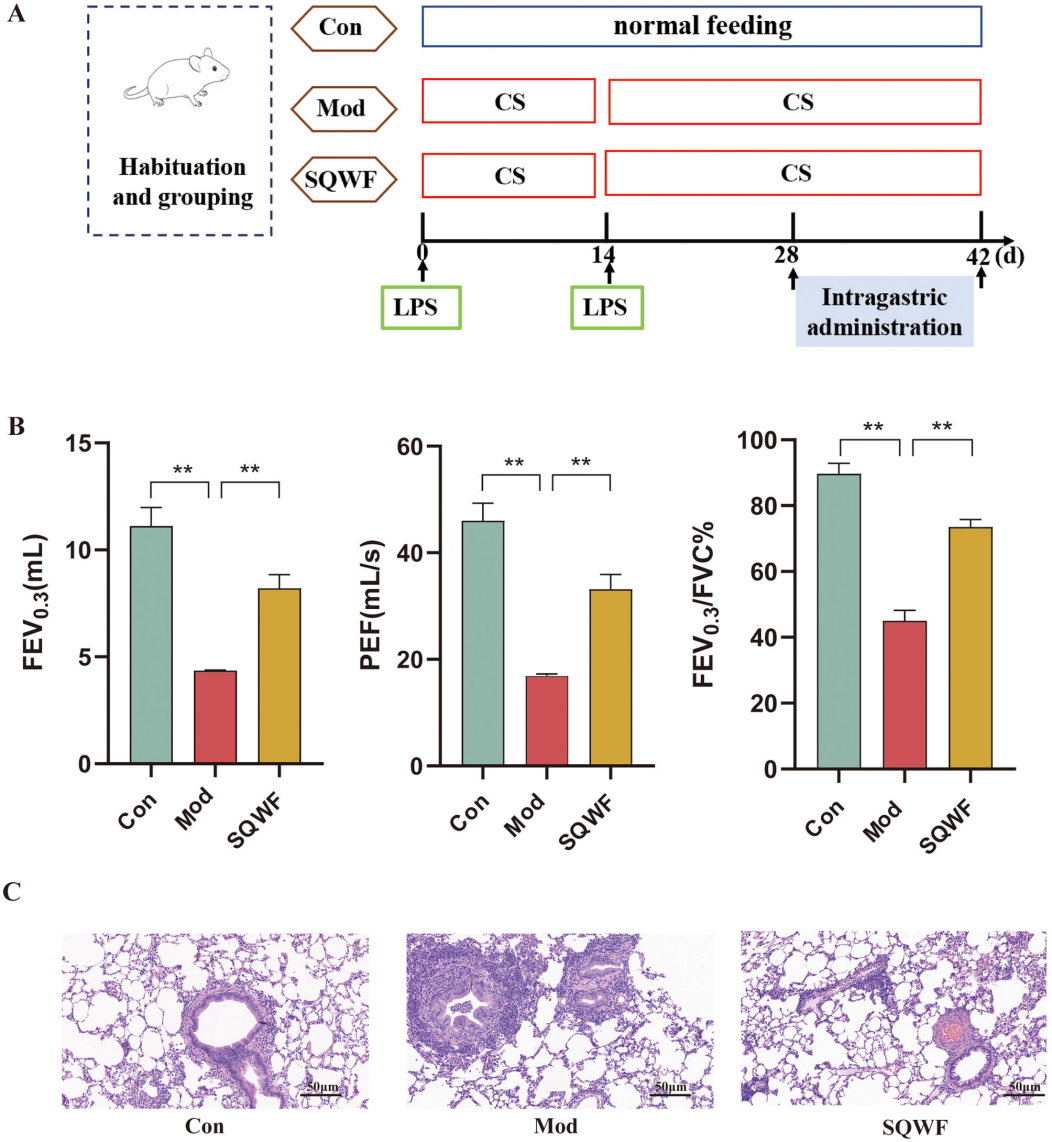
Timeline of rat modeling and drug administration **(A)**. Lung function indicators including FEV0.3, FEV 0.3/FVC, and PEF, in the different groups **(B)**; Structures of lung tissues and pathological indices in the different groups; HE staining, 20× magnification **(C)**. *n* = 3; values are expressed as mean ± SD, **p < 0.01.

Lung function assessment has been the gold standard for the diagnosis of COPD. As shown in Figure 2B, rats in the Mod group had significantly lower FEV0.3, FEV0.3/FVC, and PEF values than rats in the Con group (*p* < 0.01), while SQWF intervention improved these values compared to the Mod rats (*p* < 0.05). This indicates that the combination of CS and LPS induced a significant reduction in lung function in the rats, which was mitigated by SQWF treatment. HE staining of histological sections showed normal alveolar morphology in the Con group, characterized by well-formed and intact alveolar structures, the absence of congestion or edema in the mucosa or airway walls, and a minimal presence of inflammatory cells. In the Mod group, the alveolar cavities were enlarged and abnormally shaped, with disrupted and fused alveolar walls. The airway lumen was narrowed, accompanied by an accumulation of inflammatory cells in and around the airway walls. Further, the thickening of the airway walls was evident, along with the sloughing of some epithelial cells. Compared with the Mod group, tissues in the SQWF group showed enlargement of alveolar spaces with some irregular arrangement, and small amounts of mucosal epithelial cell sloughing, inflammatory cell infiltration, and edema (Figure 2C).

### 3.2 Analysis of alpha and beta diversity of rat intestinal microbiota

**Figure 3:**
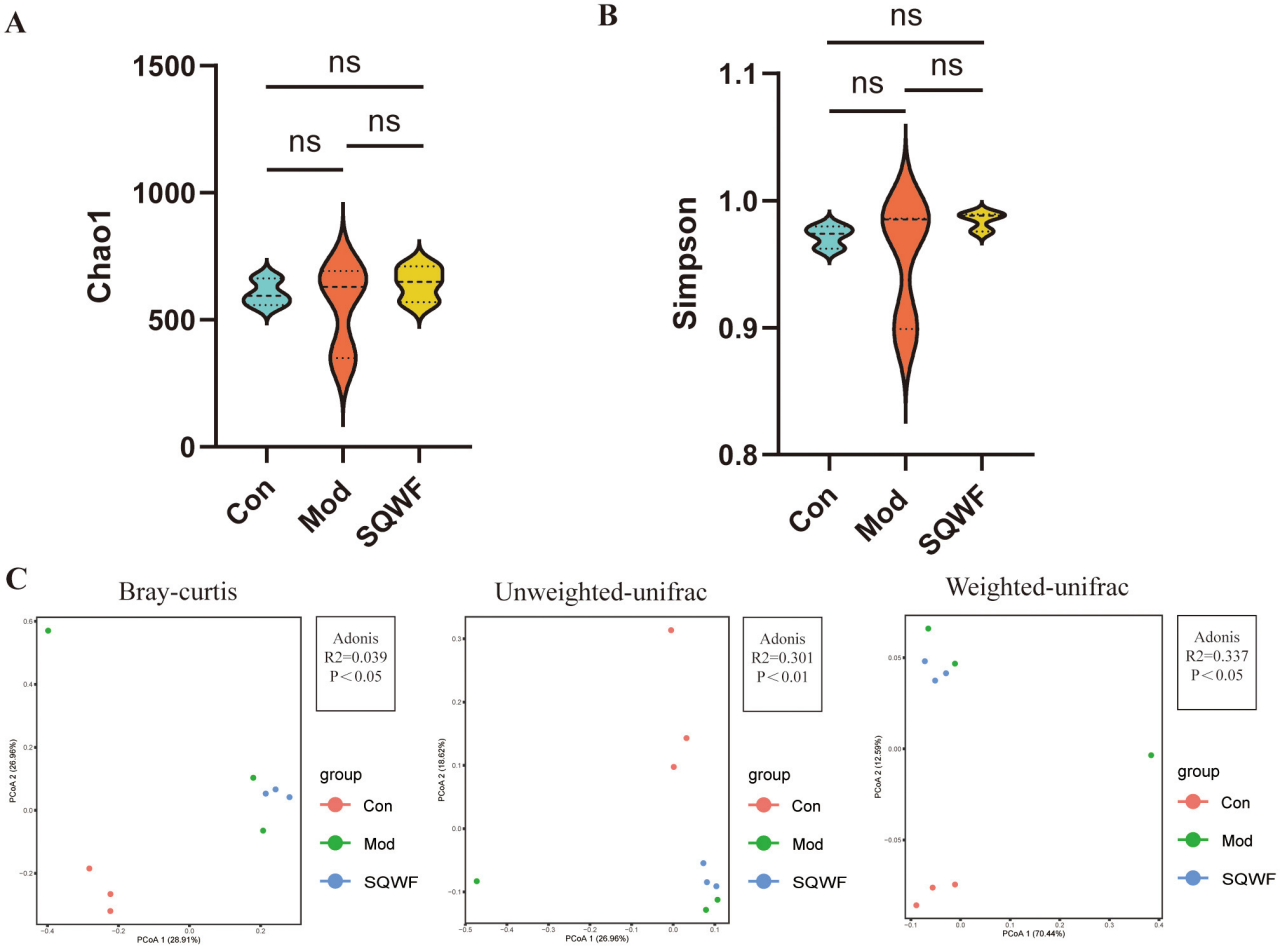
Diversity analysis of rat intestinal contents. Assessment of gut microbial richness and diversity using the Chao1 and Simpson indices **(A, B)**. PCoA plot based on Bray-Curtis dissimilaries, unweighted UniFrac, and weighted UniFrac **(C)**. Data are expressed as mean ± standard deviation (SD). *n* = 3, *p* < 0.05, the difference is statistically significant; ns, the difference is not statistically significant.

Since intestinal microorganisms play an important role in the pathogenesis of COPD, the microbiota of rats in the three groups were analyzed to assess changes in the intestinal flora of COPD model rats and the effects of SQWF intervention. The gut contents of the rats were analyzed by 16S rDNA sequencing. First, the Chao1 and Simpson indices were used to compare the alpha diversity of the intestinal microbiota in the different groups. The Chao1 index showed that the species abundance in the Con, Mod, and SQWF groups was similar (Figure 3A), while the Simpson index assessment revealed comparable species evenness across the three groups (Figure 3B). Beta diversity analysis using PCoA plots based on Bray-Curtis dissimilarities, unweighted UniFrac, and weighted UniFrac, showed significant differences in intestinal flora among the three groups. The results demonstrated a statistically significant difference in total diversity, indicating that the composition of intestinal microorganisms varied significantly among the three groups (Figure 3C).

### 3.3 Composition of intestinal flora after SQWF intervention

**Figure 4:**
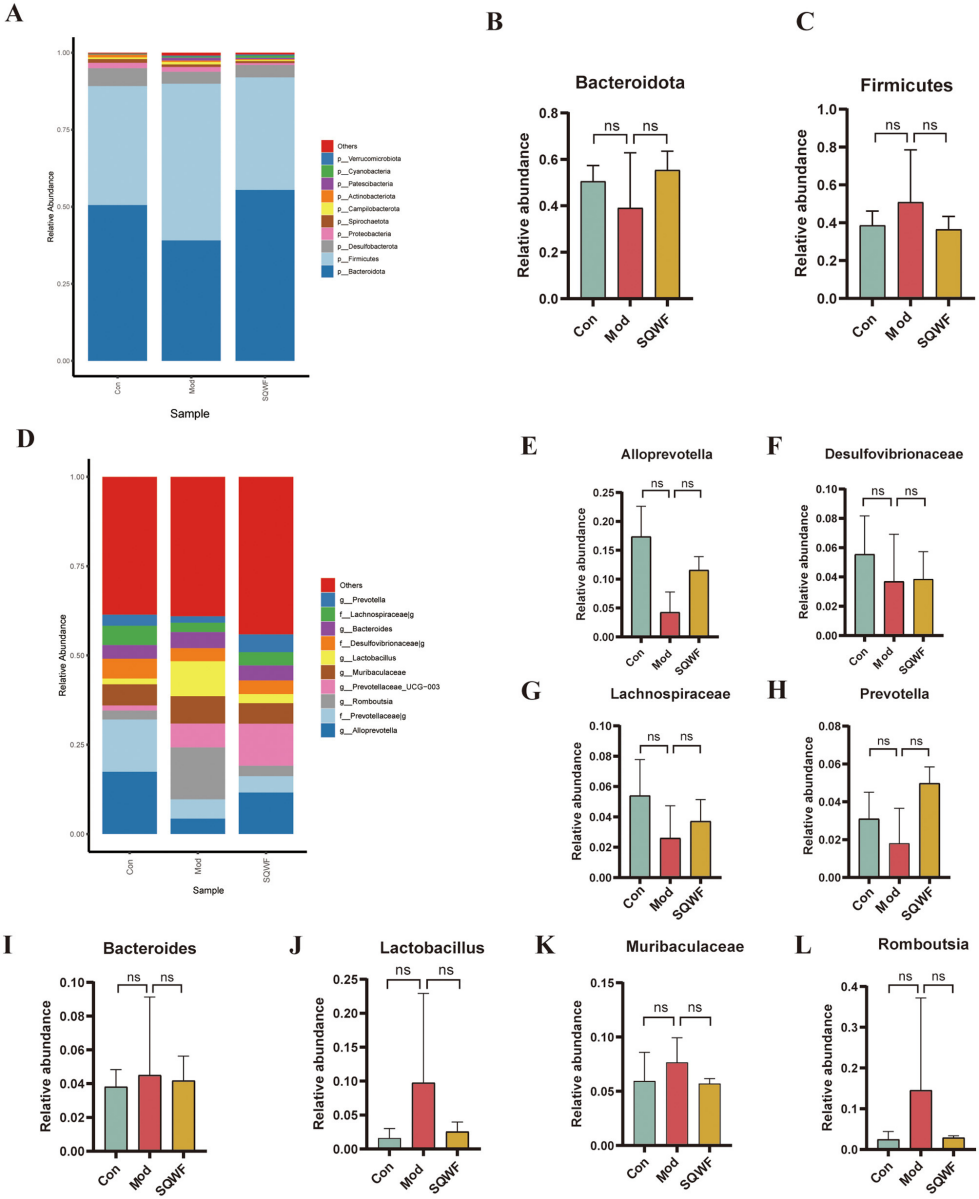
Relative abundance of gut microbiota at the phylum and genus levels. Relative abundance of major bacterial phyla **(A)**. Relative abundance of Bacteroidota and Firmicutes phyla **(B, C)**. Relative abundance of major bacterial genera **(D)**. Relative abundance of *Alloprevotella, Desulfovibrionaceae, Lachnospiraceae, Prevotella, Bacteroides, Lactobacillus, Muribaculaceae*, and *Romboutsia* genera **(E–L)**. Data are expressed as mean ± standard deviation (SD).

To further demonstrate the effect of SQWF on the composition of the intestinal microbiota in COPD rats, the top 10 microorganisms in each group were identified at the phylum and genus levels. Analysis of the microbial community structures at the phylum level showed that the dominant bacterial phyla in the three groups included *Bacteroidota, Firmicutes, Desulfobacterota, Proteobacteria* and *Spirochaetota, Campilobacterota, Actinobacteriota, Patescibacteria, Cyanobacteria*, and *Verrucomicrobiota* (Figure 4A). The intestinal microorganisms were dominated by *Bacteroidota* and *Firmicutes* (Figures 4B, C). The results showed that the abundance of the intestinal microorganisms differed among the three groups at the phylum level.

At the genus level, the top 10 most abundant intestinal microorganisms included *Alloprevotella, Prevotellaceae_g-, Romboutsia, Prevotellaceae_UCG-003, Muribaculaceae, Lactobacillus, Desulfovibrionaceae_g-, Bacteroides, Lachnospiraceae_g-*, and *Prevotella* (Figure 4D). Compared with the control group, the relative abundance of *Alloprevotella, Desulfovibrionaceae_g-, Lachnospiraceae_g-*, and *Prevotella* was reduced in the Mod group but increased after SQWF intervention (Figures 4E–H). Compared with the control group, the relative abundance of *Bacteroides, Lactobacillus, Muribaculaceae*, and *Romboutsia* was increased in the Mod group and decreased after SQWF intervention (Figures 4I–L). The results that, although the changes in the relative abundance of intestinal microorganisms among the three groups were not statistically significant at the genus level, treatment with SQWF resulted in an overall normalization of the bacterial flora.

### 3.4 Differences in the intestinal flora after SQWF intervention

**Figure 5:**
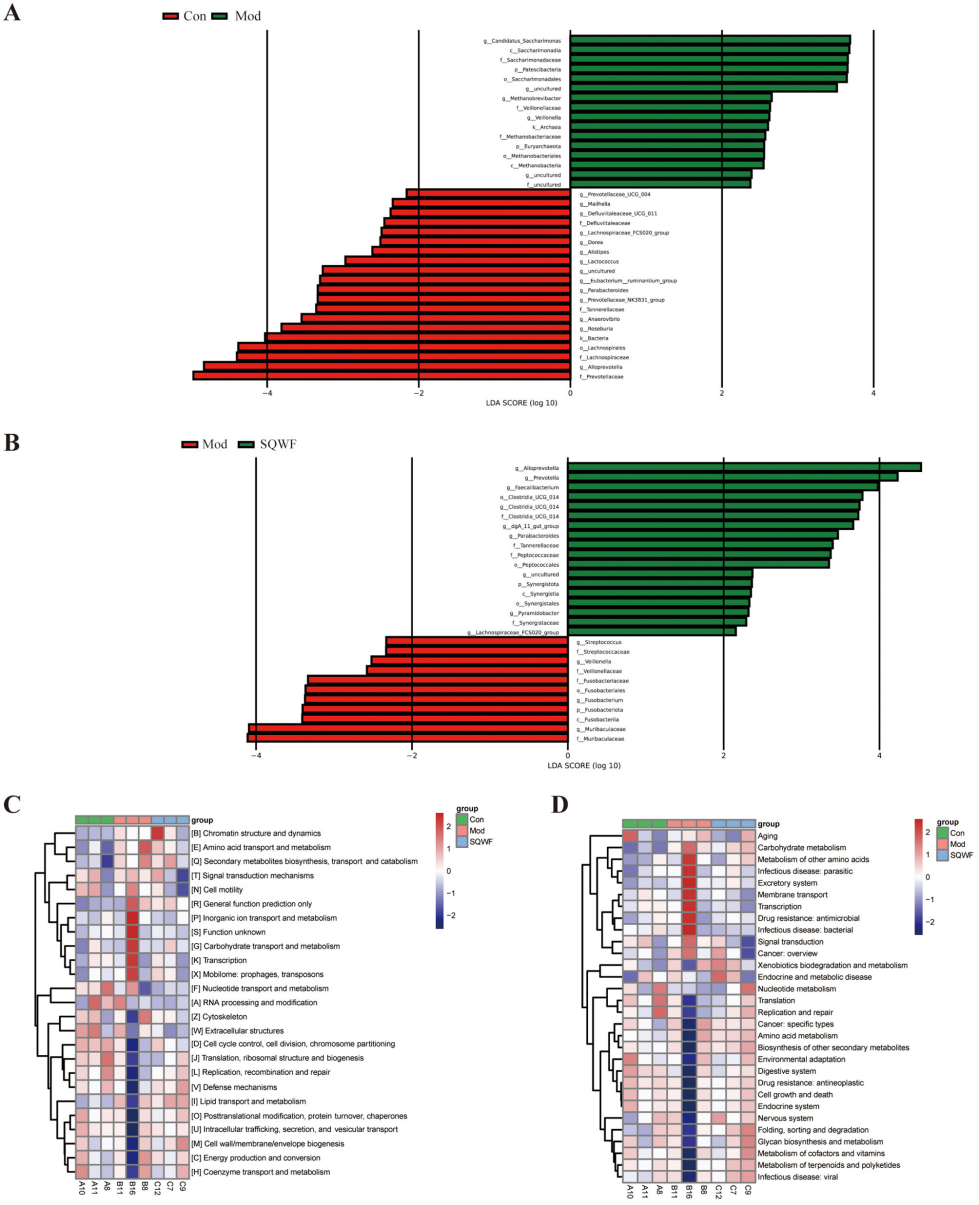
Differences in core bacterial phenotypes and functional predictions of bacterial communities by PICRUSt2. Microbial groups showing differential abundance between the Con and Mod groups **(A)**. Microbial groups showing differential abundance between the Mod and SQWF groups **(B)**. COG functional predictions of differentially abundant bacteria **(C)**. KEGG functional predictions of differentially abundant bacteria **(D)**. |LDA| > 2 was used as the criterion for differential abundance.

LEfSe analysis was used to identify microorganisms showing differential abundance among the three groups. This indicated that 16 16 microbial taxa were enriched in the intestinal contents of rats in the Mod group compared with the Con group, while the abundance of 20 taxa was reduced in the Mod group relative to the Con group (Figures 5A, B). Compared with the Mod group, the relative abundance of 11 taxa was decreased following treatment with SQWF, while the abundance of 18 taxa was increased (Figure 4B). It is worth noting that compared with the control group, *g_Alloprevotella, g_Parabacteroides, f_Tannerellaceae*, and *g_Lachnospiraceae_FCS020* were reduced in the Mod group but were up-regulated after SQWF intervention (Figures 5A, B). However, compared with the Con group, *f_Veillonellaceae*, and *g_Veillonella* were more abundant in rats in the Mod group and decreased after SQWF treatment (Figures 5A, B). These results indicate that the intestinal flora was disrupted in COPD rats compared with normal rats, while these changes were alleviated by treatment with SQWF.

### 3.5 Functional prediction of target genes influenced by SQWF treatment

Functional prediction using the COG database showed that genes associated with COPD induction in rats in the Mod group were associated with significant enrichment in chromatin structure and dynamics, amino acid transport and metabolism, secondary metabolite biosynthesis, transport and catabolism, signal transduction mechanisms, cell motility, general function prediction only, inorganic ion transport and metabolism, carbohydrate transport and metabolism, transcription, and mobilome, prophages, and transposons. These enrichments were significantly reduced after SQWF treatment (Figure 5C).

KEGG analysis showed that the gut microbiota of rats in the Mod group was significantly associated with pathways involved in the metabolism of other amino acids, infectious disease: parasitic, excretory system, membrane transport, infectious disease: bacterial, drug resistance: antimicrobial, transcription, signal transduction, carbohydrate metabolism, and cancer: overview, relative to the Con group. These enrichments were significantly reduced after SQWF treatment (Figure 5D).

### 3.6 The effects of SQWF predict key targets for the treatment of COPD through the lung-gut axis

**Figure 6:**
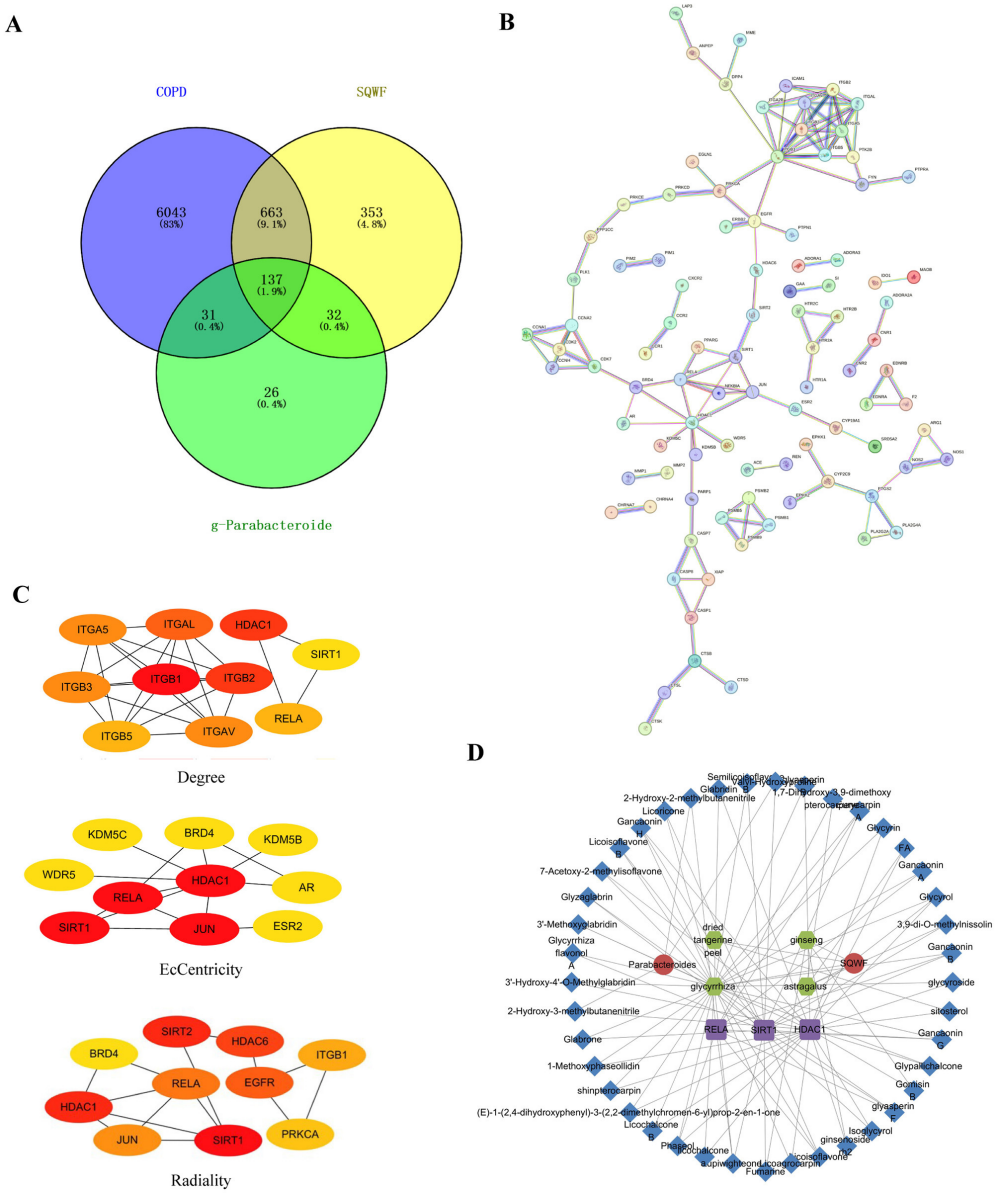
SQWF improves network pharmacological outcomes of COPD by regulating microbiota metabolism. Venn diagrams were used to identify overlapping COPD disease targets, SQWF targets, and intestinal flora metabolic targets **(A)**. PPI network of the overlapping genes, constructed by STRING **(B)**. The top 10 genes identified **(C)**. PPI network showing traditional Chinese medicine compound-bacteria-metabolites-targets. The red circles represent SQWF and bacteria *g-Parabacteroides*, green circles represent active ingredients, purple squares represent core gene targets, and blue diamonds represent metabolites **(D)**.

To predict the key targets associated with SQWF treatment of COPD through the lung-gut axis, genes in the intersection of the COPD disease targets, SQWF drug targets, and metabolic targets of intestinal flora were analyzed. First, 6874 COPD disease-related targets were identified using GeneCards, OMIM, and DisGeNET databases. Compounds included in the various traditional Chinese medicines in SQWF were screened based on TCMSP. Duplicate compounds were removed, and the corresponding SMILES names were searched in the PubChem database, while the STP database was used to identify potential targets of the compounds. After eliminating duplicate targets, a total of 1,185 targets were obtained. Based on the results of the LEfSe analysis (|LDA| > 2, Figures 5A, B), four beneficial bacteria *g-Alloprevotella, g-Parabacteroides, g-Lachnospiraceae* FCS020 group, and *f-Tannerellaceae*, were obtained and entered into the gutMGene database. Three compounds corresponding to *Parabacteroides* were used to predict the targets using the PubChem and STP databases, and 226 targets were identified after deduplication. The overlap between drug/bacteria/disease targets identified 137 genes (Figure 6A). The PPI network of these overlapping genes is shown in Figure 6B, and the top 10 core genes identified by CytoHubba are shown in Figure 6C. Three significant hub genes, ReLA, SIRT1, and HDAC1, were found. The PPI network of the traditional Chinese medicine compound-intestinal bacteria-metabolites-targets is illustrated in Figure 6D.

### 3.7 Enrichment analyses of overlapping genes

**Figure 7:**
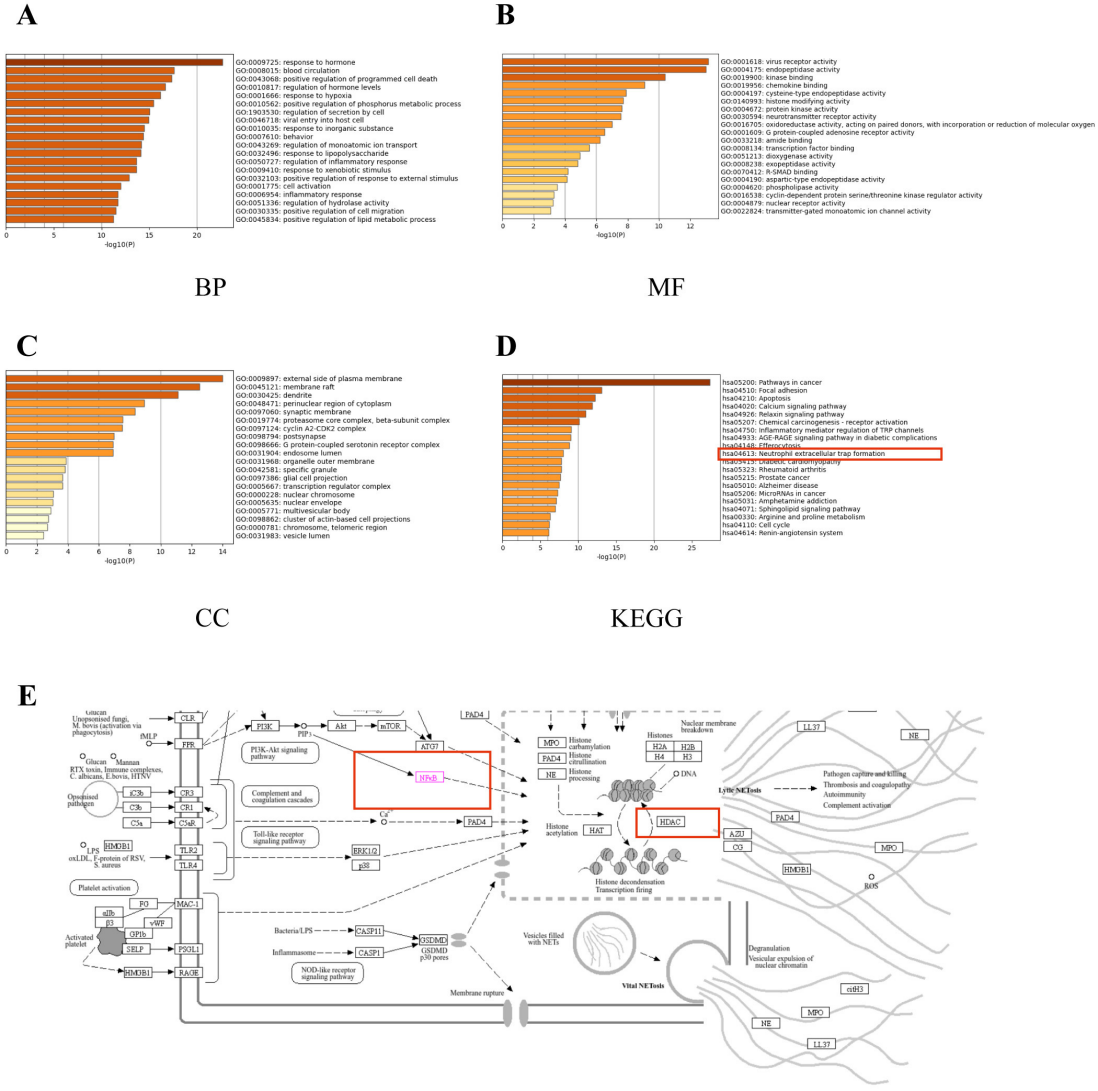
Enrichment analyses of overlapping genes. The top 20 GO molecular function (MF) enrichments **(A)**. The top 20 GO biological process (BP) enrichments **(B)**. The top 20 GO cellular component (CC) enrichments **(C)**. The top 20 KEGG pathway enrichments **(D)**. Thumbnail image of neutrophil extracellular trap formation derived from the KEGG Pathway Database **(E)**. Gene ratio = number/set size.

To further explore the functions of the overlapping genes, GO and KEGG enrichment analyses were performed, identifying the top 20 items in the GO categories of biological process (BP), molecular function (MF), and cellular component (CC), as well as the relevant KEGG pathways (Figures 7A–D). The GO BP terms included positive regulation of programmed cell death, response to hypoxia, positive regulation of phosphorus metabolic process, and regulation of secretion by cell (Figure 7A), while the MF terms were virus receptor activity, endopeptidase activity, kinase binding, chemokine binding, and cysteine-type endopeptidase activity (Figure 7B) and the CC terms included the external side of plasma membrane, membrane raft, dendrite, perinuclear region of cytoplasm, synaptic membrane, cyclin and A2-CDK2 complex (Figure 7C). The KEGG analysis showed enrichment in pathways associated with Apoptosis, calcium signaling pathway, relaxin signaling pathway, receptor activation, inflammatory mediator regulation of TRP channels, and neutrophil extracellular trap formation (Figure 7D). Combining the core genes calculated by CytoHubba with the KEGG analysis led to the identification of three core genes, ReLA, SIRT1, and HDAC1 (Figure 6C). Furthermore, both ReLA and HDAC1 can regulate the neutrophil extracellular trap formation signaling pathway (Figure 7E). Therefore, we verified the regulatory effects of ReLA and HDAC1 on NETs.

### 3.8 Molecular docking of SQWF active ingredients and core target proteins

**Figure 8:**
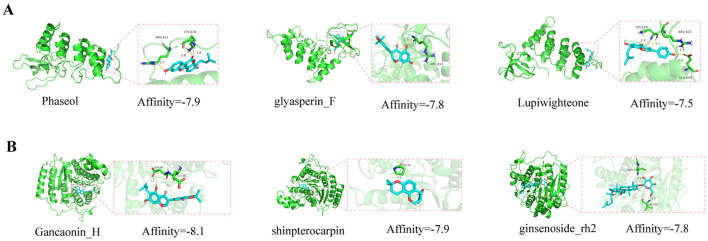
Molecular docking model diagrams. Molecular docking of SQWF active ingredients and RelA **(A)**. Molecular docking of SQWF active ingredients and HDAC1 **(B)**.

Molecular docking of the key active components of SQWF was performed with the RelA and HDAC1 structures. This showed that six active ingredients were bound to RelA and 24 active ingredients bound to HDAC1. In particular, the docking binding energies of RelA with Phaseol, glyasperin_F, and Lupiwighteone were −7.9, −7.8, and −7.5 kcal/mol, respectively (Figure 8A), showing strong binding activity. Similarly, the docking binding energies of HDAC1 with Gancaonin_H, shinpterocarpin, and ginsenoside_rh2 were −8.1, −7.9, and −7.8 kcal/mol, respectively (Figure 8B), indicating strong binding activity.

### 3.9 SQWF regulates the expression of hub genes

**Figure 9:**
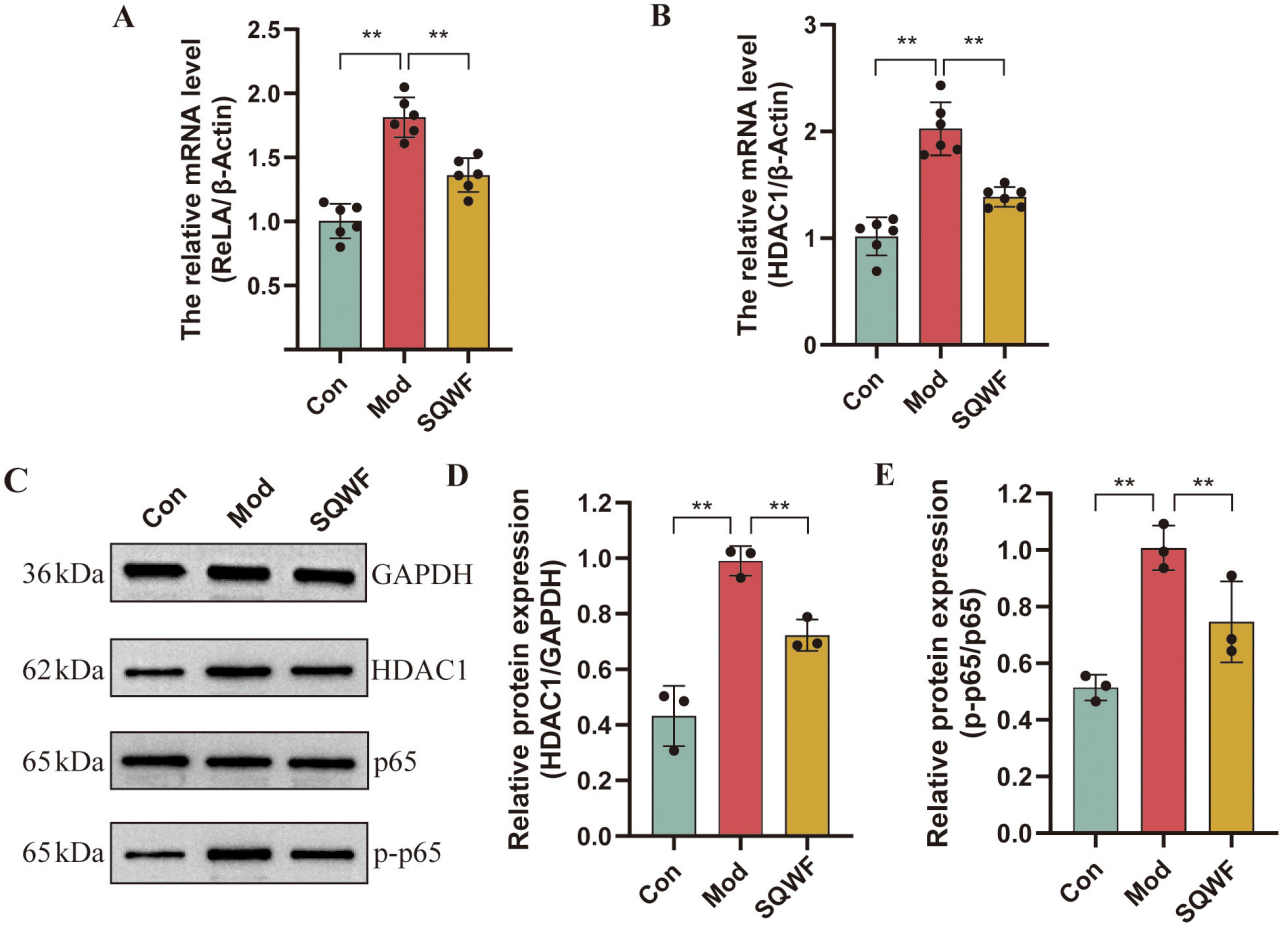
Expression of *RELA* and *HDAC1* in rat lung tissue. Relative expression of *RELA* **(A)** and *HDAC1* mRNA in lung tissue, *n* = 6 **(B)**; Western blotting of p65, p-p65, and HDAC1 protein expression in rat lung tissue, *n* = 3 **(C–E)**. Values are expressed as mean ± SD. **p < 0.01.

The expression of core genes and proteins in rat lung tissue was further investigated. As shown in Figures 9A–B, rats in the Mod group showed higher levels of ReLA and HDAC1 mRNA expression in their lung tissues compared to the Con group (*p* < 0.01), while compared with the Mod group, the levels of ReLA and HDAC1 mRNA in the SQWF group were decreased (*p* < 0.01). The phosphorylation of p65 was then examined (Figures 9C–E), finding that the relative expression of p-p65/p65 in the lung tissue of rats in the model group was increased (*p* < 0.01). However, when compared with the Mod group, the p-p65/p65 levels in the SQWF group were reduced (*p* < 0.01).

### 3.10 SQWF reduces the formation of NETs in rat lung tissue

**Figure 10:**
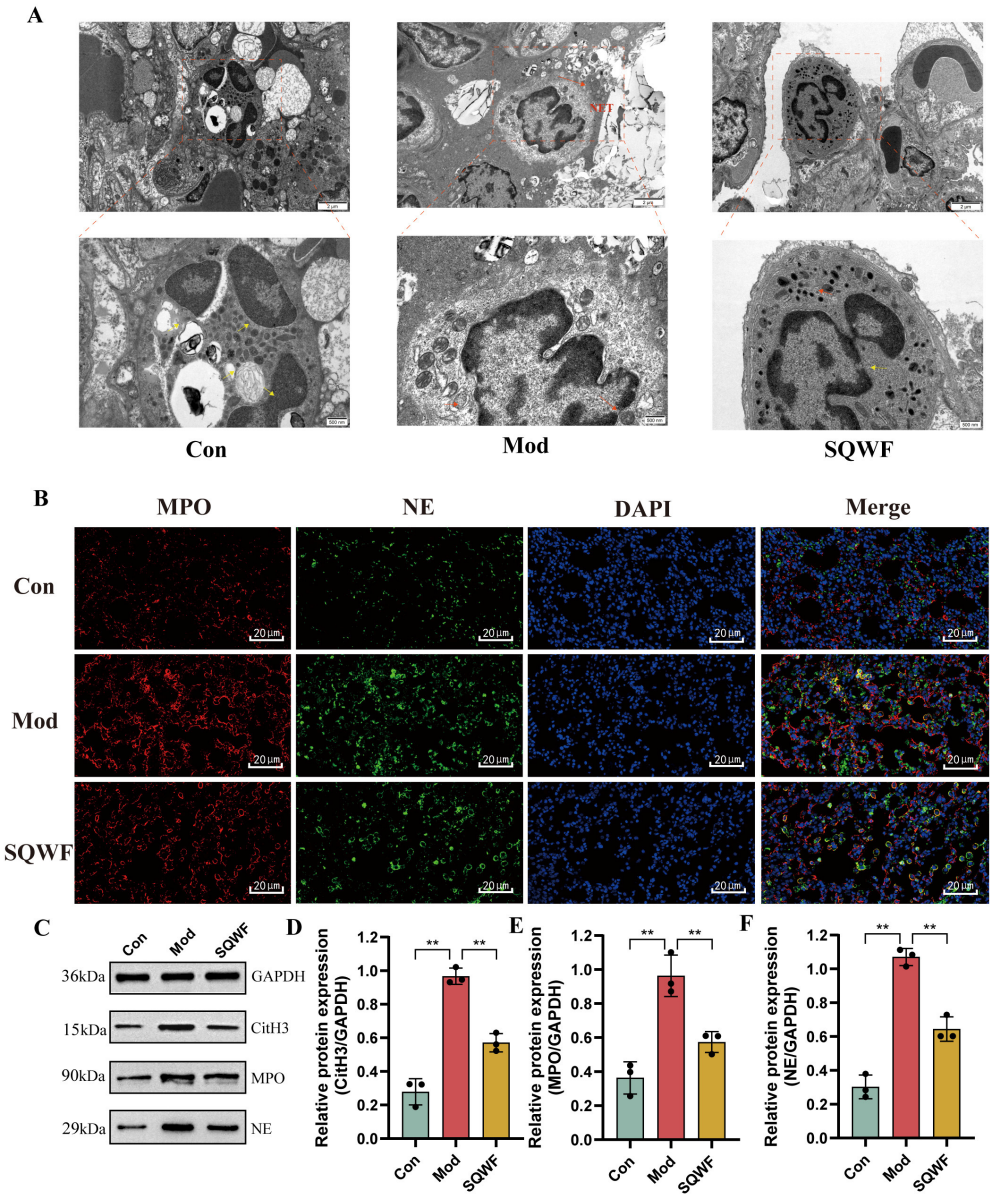
NET production and expression of associated proteins in rat lung tissues. The effect of SQWF on the ultrastructure of lung tissue induced by CS (10,000× and 25,000×); the yellow arrow indicates normal morphologicy and the red indicates abnormal morphologicy **(A)**. Representative confocal microscopy images of immunofluorescence staining (blue, DAPI; green, NE; red, MPO) showing colocalization of neutrophil nuclei with NE and MPO in the lung tissue of rats in the Mod group. SQWF treatment prevents NE nuclear translocation **(B)**. Western blotting showing the expression of NE **(C)**, MPO **(D)**, and CitH3 **(E)** in rat lung tissue. *n* = 3. Values are expressed as mean ± SD. **p < 0.01.

The neutrophil status in the lung tissues of rats in the different groups was assessed using TEM. In the Con group, the neutrophils showed normal morphology, with multi-lobed nuclei. A large number of mitochondria were observed in the cytoplasm, which was oval, with clear internal cristae and intact cell membranes without rupture. Abnormal neutrophil morphology was seen in the Mod group, with the widening of the nuclear membrane space, nuclear pyknosis, disappearance of lobular morphology, and cell membrane rupture, forming NETs. In contrast, the morphology of the neutrophils in the SQWF group was relatively normal. The nuclei were multi-lobed, and while a small number of mitochondria in the cytoplasm were swollen, the cell membrane was essentially intact without rupture (Figure 10A).

Immunofluorescence was used to visualize the co-localization of MPO and NE. As shown in Figure 10B, in the Con group, NE and MPO were mostly localized in cytoplasmic granules, with relatively low expression levels. In contrast, in the Mod group, diffuse DNA staining of neutrophils co-localized with MPO and NE. It is worth noting that SQWF treatment prevented the nuclear translocation of both NE and MPO, resulting in them remaining in the cytoplasm.

NET markers include NE, CitH3, and MPO (Brinkmann et al., 2004; Papayannopoulos et al., 2010; Douda et al., 2015). Thus, the presence of NETosis based on the expression of these proteins was examined in rat lung tissue. Compared with the normal group, the protein expression levels of NE, MPO, and CitH3 were increased in lung tissue from rats in the Mod group but decreased significantly after treatment with SQWF (*p* < 0.01, Figures 10C–F). This indicates that SQWF can improve CS-induced NETosis in lung tissue.

## 4 Discussion

The largest microbiota in the human body is found in the intestine, associated with persistent and rapidly renewing epithelial cells. The intestinal flora and the host have formed a close symbiotic relationship over an extensive evolutionary history. Homeostasis between the host and the intestinal microbiota is critical for maintaining a healthy intestinal barrier and optimal immune status, effectively preventing and reducing the occurrence of disease (Xu and Gordon, 2003; Chakradhar, 2017; Wang et al., 2018). Several studies have now shown an interaction between the lungs and intestines. The close connection between the organs of the lung-gut axis may originate from their similar physiological structures. Microvilli and cilia form the physical barriers of the intestine and respiratory tract, respectively, and they participate in local immune responses together with lymphoid tissue (Espirito Santo et al., 2021). Short-chain fatty acids can reduce airway inflammation and protect patients with airway inflammation, indicating that the metabolites of the intestinal flora can regulate lung function through immune responses (Cait et al., 2018). In addition, the transplantation of anti-inflammatory intestinal microorganisms from mice into wild-type mice can inhibit CS-induced lung inflammation (Nascimento et al., 2023). It was also demonstrated that the phagocytic function of alveolar macrophages against *Streptococcus pneumoniae* was also demonstrated to be reduced in mice with intestinal microbial depletion (Schuijt et al., 2016). This evidence suggests that gut microbes are critical for controlling lung inflammation. Based on this, this study focused on imbalances in the intestinal flora caused by COPD and found that SQWF may have a protective effect on COPD by improving the intestinal flora.

The 16S rDNA results showed that the species composition of the intestinal microorganisms of the three groups of rats differed significantly. Compared with the Con group, changes in the intestinal flora in the Mod group were primarily reflected by reductions in the relative abundance of *Prevotella* and *Bacteroidota*, and increased relative abundance of *Firmicutes*. Although *Prevotella*, a plant-based dietary probiotic species, has not been reported to be associated with healthy lungs, its abundance was reduced in COPD mouse models (Larsen, 2017; Liu et al., 2024). *Bacteroidota* and *Firmicutes* are the cornerstone bacteria of the intestinal tract. Previous experimental results have shown that an increase in the Firmicutes/Bacteroidota ratio leads to increased production of inflammatory and genotoxic substances. The present research found that the imbalance in these bacterial genera was alleviated after SQWF treatment. Therefore, the protective effect of SQWF on the COPD rat model may derive from its ability to alleviate imbalances in the gut microbiota.

In addition, LEfSe analysis was used to identify microbial communities with significant differences between the different groups. Compared with the Con group, the bacterial genera that were significantly reduced in COPD rats included *Lachnospiraceae_FCS020_group, Alloprevotella*, and *Parabacteroides*. *Lachnospiraceae* is a potential probiotic intestinal microorganism and studies have found that its abundance is negatively correlated with the levels of pulmonary inflammatory factors (Wu X. et al., 2023). *Alloprevotella* is positively correlated with lung function levels in patients with COPD, which may be related to the anti-inflammatory effect of short-chain fatty acids (SCFAs), such as acetic acid, produced by fermentation of *Alloprevotella* (Zhao et al., 2024). *Parabacteroides* are core members of the human intestinal microbiota and can promote the expression of anti-inflammatory T cell phenotype CD4 + CD25 + T cells and IL-10 + FoxP3 + regulatory T cells (Cekanaviciute et al., 2017). Lai et al. (2022) have found that *Parabacteroides* may counteract inflammation and metabolic abnormalities by inhibiting TLR4 signaling, thereby improving the pathological morphology and lung function of COPD lungs. In this study, the levels of these bacterial flora were found to normalize after SQWF intervention, indicating that SQWF may alleviate the chronic inflammation of the lungs in COPD by regulating the levels of these bacteria.

Changes in the composition of the intestinal flora can induce changes in the host's metabolic phenotype, thereby indirectly leading to disease progression. A previous study demonstrated that butyrate produced by probiotics such as *Ruminococcus* can enhance the functions of both the intestinal barrier and the lungs, while also inhibiting the inflammatory response (Wang et al., 2023). Disturbances in the intestinal flora have been shown to lead to a decrease levels of docosatetraenoic acid, and several studies have reported a positive correlation between docosatetraenoic acid and lung function in smokers (McKeever et al., 2008; Zhou et al., 2023). *Parabacteroides* inhibit lung inflammation in COPD by restoring amino acid metabolites such as proline in the serum (Lai et al., 2022). These results suggest that the intestinal flora may regulate metabolite levels in COPD, thus affecting the progression of the condition.

Inhibiting the production of neutrophil extracellular traps (NETs) is one of the important mechanisms by which the intestinal flora regulates inflammation. It has been reported that *R. intestinalis, Bacteroides*, and their associated metabolite butyric acid inhibit inflammation and alleviate histopathological damage by reducing the formation of NETs (Tian et al., 2022; Li et al., 2023). NETs are large reticulated structures that bind pathogens, inhibit microbial transmission, and destroy bacterial virulence factors. Cigarette smoke extract (CSE) has been shown to induce NET formation in an NADPH oxidase-dependent manner, targeting macrophages and human bronchial epithelial (HBE) cells, and potentially important pathways for chronic airway inflammation in COPD (Zou et al., 2020). However, there are currently no studies investigating the inhibition of NETs in the lungs mediated by the lung-gut axis by the gut microbiota. This study found that SQWF can regulate imbalances in the intestinal flora in rats. Through online database prediction and combined with network pharmacology, it was determined that SQWF can improve COPD by regulating the metabolites of the probiotic *Parabacteroides*. These metabolites mainly target HDAC1 and RelA and function by regulating the NET signaling pathway. Upregulation of RelA, a subunit of the NF-κB transcription complex, was associated with decreased abundance of Parabacteroides (Briviba et al., 2023). RelA has been reported to regulate inflammation and apoptosis in COPD (Gao et al., 2024). Meanwhile, SCFAs produced by Parabacteroides are inhibitors of HDAC1 (Yan et al., 2022; Hu et al., 2024), which has been reported to be associated with macrophage polarization in COPD (Zhang et al., 2023). Similarly, the molecular docking results indicated that the active ingredients in SQWF bound effectively to the target proteins. The *in vivo* experiments also confirmed that SQWF can alleviate COPD by regulating the probiotic *Parabacteroides* through targeting HDAC1 and RelA to modulate the NET signaling pathway.

NETs in the airways of patients with COPD are associated with exacerbation frequency and deteriorations in lung function (Grabcanovic-Musija et al., 2015; Dicker et al., 2018). When stimulated by LPS, bacteria, cigarette smoke, and some environmental factors, the NE and MPO translocate to the nucleus, leading to chromatin decondensation and NET formation. This in turn leads to the rupture of the plasma membrane and the death of the cell (Burns et al., 2003; Papayannopoulos et al., 2010). CitH3, NE, MPO, and DNA elastase, among others, are characteristic components of NETs (Wang et al., 2009; Papayannopoulos et al., 2010; Akong-Moore et al., 2012; Lewis et al., 2015). Increased expression of the NET-related proteins NE, MPO, and citH3 is reported to promote both inflammation and tissue damage (Jiang et al., 2013; Thulborn et al., 2019). The present results indicate that treatment with SQWF can reduce the expression of NE, MPO, and CitH3 in the lungs of COPD model rats, thereby decreasing NET production. This suggests that SQWF may alleviate lung inflammation and mitigate lung tissue damage by inhibiting the production of NETs.

NF-κB plays an important role in the process of NET formation. Studies have shown that neutrophil stimulation promotes the nuclear translocation of NF-κB/RelA(p65) proteins and degradation of IkB-α, promoting NET formation and subsequent inflammation. HDAC1 is a member of the class I HDACs family. It has been shown that HDAC1 plays an important role in causing NETosis in mouse lung neutrophils by catalyzing the deacetylation of histone H3 and subsequently activating PAD4 (Poli et al., 2021). The present study found that SQWF can inhibit the expression of *RELA* and *HDAC1* genes, and reduce p65 phosphorylation, and HDAC1 protein expression, indicating that SQWF may reduce the expression of NETs in the lung tissue of rats stimulated by cigarettes by inhibiting the expression of ReLA and HDAC1.

## 5 Conclusion

This study found that SQWF can alleviate imbalances in the intestinal flora in COPD model rats, with the bacterial genus *Parabacteroides* being key to the alleviation of lung inflammation. Furthermore, the ability of *Parabacteroides* to improve COPD-associated lung inflammation through the lung-gut axis may be related to its regulation of the key target genes ReLA and HDAC1, thereby reducing the production of NETs in the lungs. In conclusion, SQWE can regulate imbalances in intestinal *Parabacteroides*, further regulating ReLA and HDAC1 expression, reducing the production of NETs in lung tissue, and thus alleviating lung inflammation (Figure 1).

## 6 Shortcomings and prospects

Several limitations of this study need to be acknowledged. Although the findings indicated that SQWF alleviated pulmonary inflammation by restoring the balance of *Parabacteroides* in the gut, thus modulating HDAC1 and ReLA expression through the lung-gut axis, and reducing NET production, the results cannot directly reflect the regulation of ReLA and HDAC1 expression by *Parabacteroides*. Further, the central roles of these two key targets in promoting NETosis in neutrophils and their interrelationship remain to be fully elucidated. Follow-up studies should focus on microbiota recovery and incorporate *in vitro* experiments utilizing lentiviral transfection and other techniques to elucidate the mechanisms by which SQWF regulates the NET signaling pathway through the modulation of *Parabacteroides* abundance.

## Data Availability

The original contributions presented in the study are publicly available. This data can be found here: https://www.ncbi.nlm.nih.gov/, accession number PRJNA1181133.
