# Supplementary figures and images for: Protective effect of Shenqi Wenfei Formula against lipopolysaccharide/cigarette smoke-induced COPD in Rat based on gut microbiota and network pharmacology analysis
Source: Front Microbiol. 2024 Nov 19;15:1441015. doi: 10.3389/fmicb.2024.1441015 (PMC11611827; doi:10.3389/fmicb.2024.1441015)

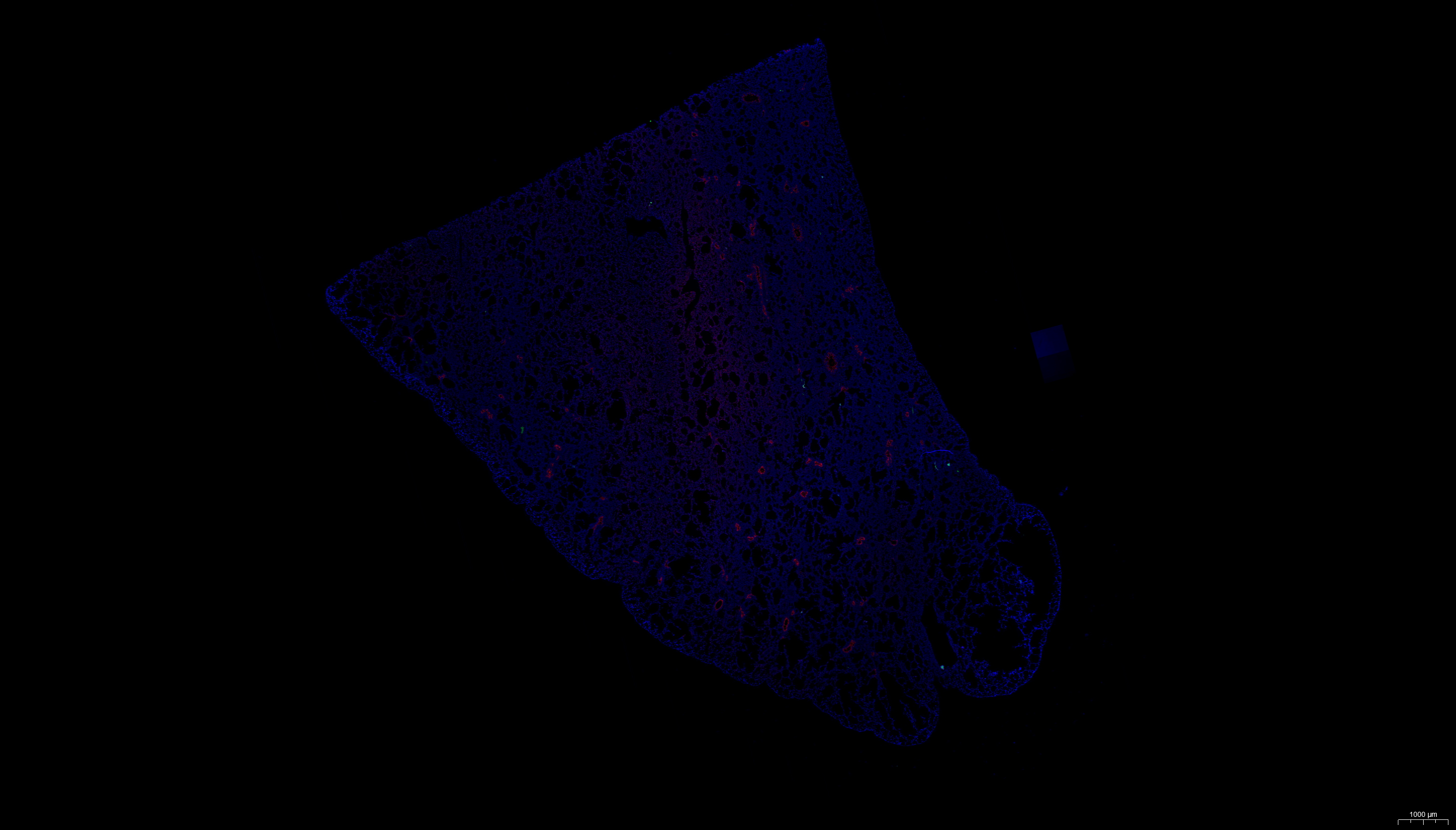

Supplement: Supplementary file 1 [file Data_Sheet_1.zip › original data1/Immunofluorescence/Con_1.0x.jpg]

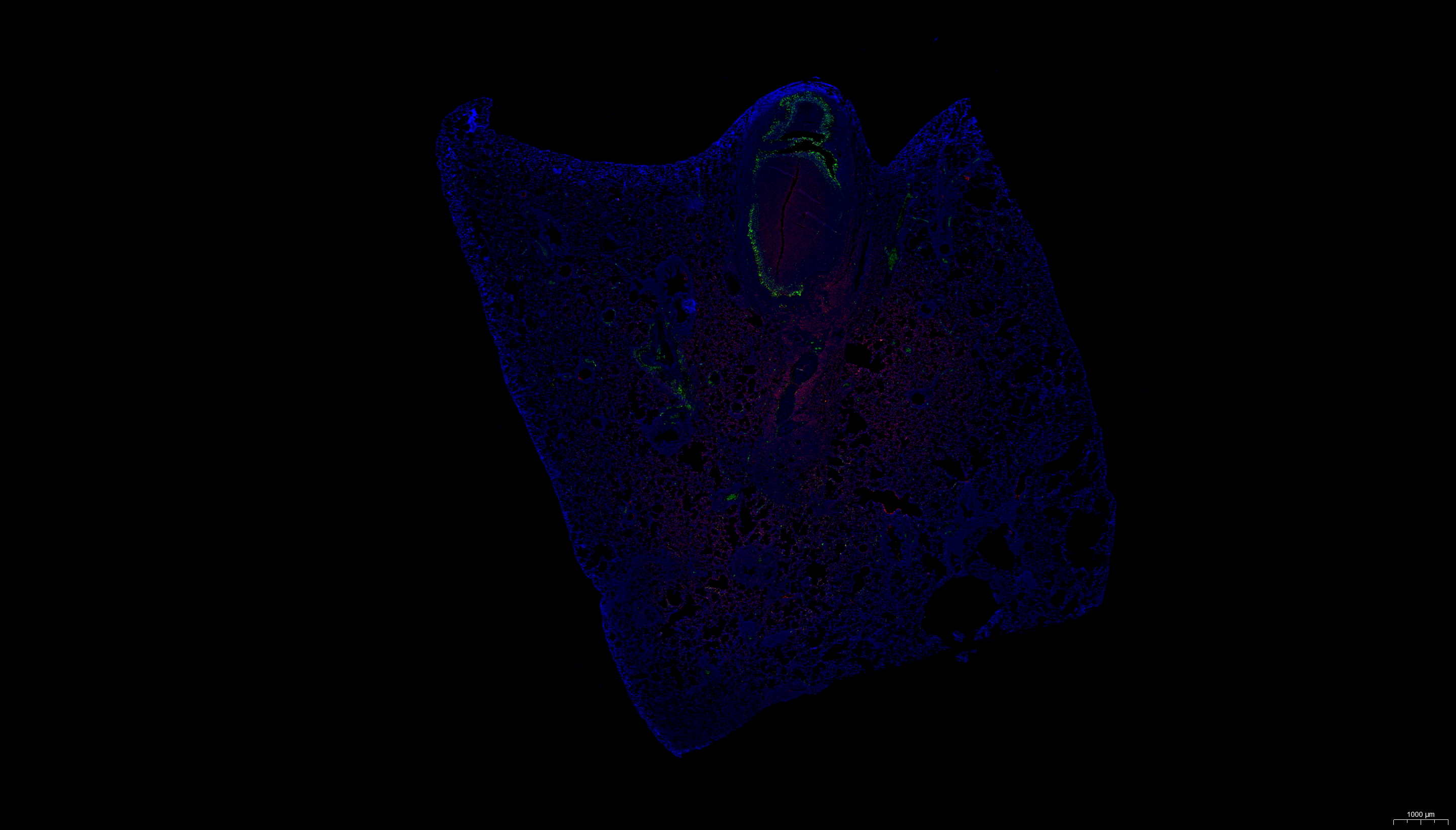

Supplement: Supplementary file 1 [file Data_Sheet_1.zip › original data1/Immunofluorescence/MOD1.1x.jpg]

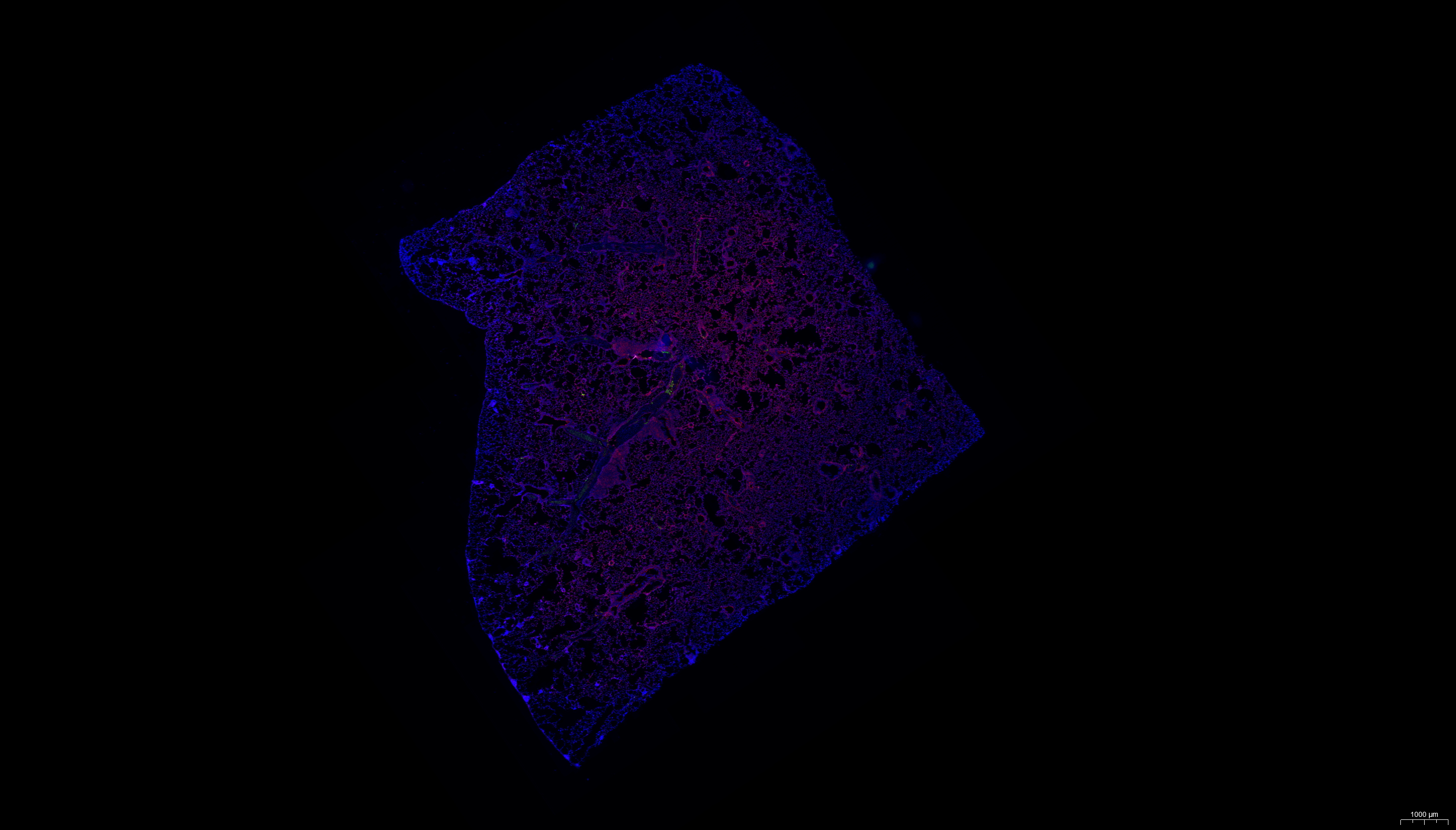

Supplement: Supplementary file 1 [file Data_Sheet_1.zip › original data1/Immunofluorescence/SQWF.jpg]

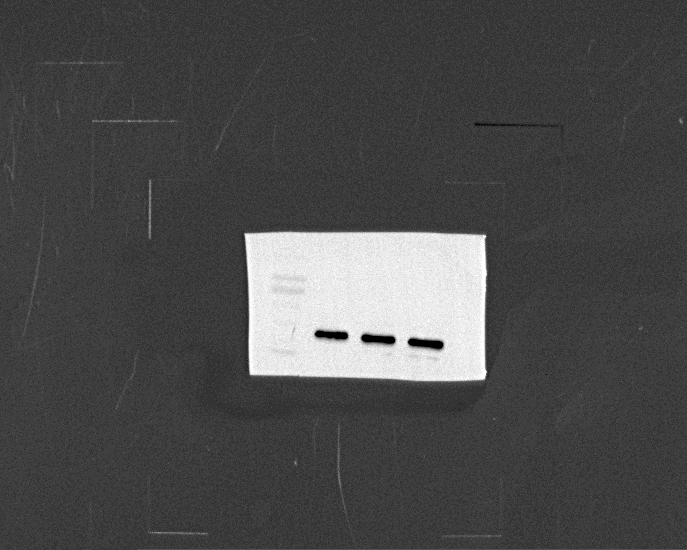

Supplement: Supplementary file 1 [file Data_Sheet_1.zip › original data1/Western blotting/WB-1/GAPDH-1.tif]

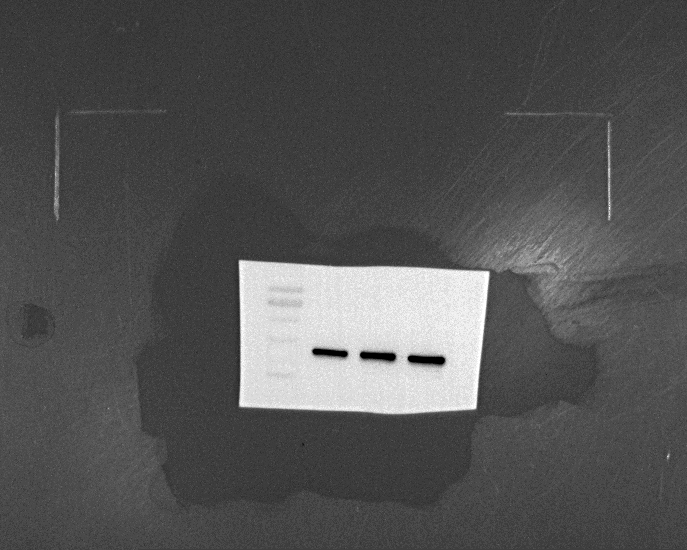

Supplement: Supplementary file 1 [file Data_Sheet_1.zip › original data1/Western blotting/WB-1/GAPDH-2.tif]

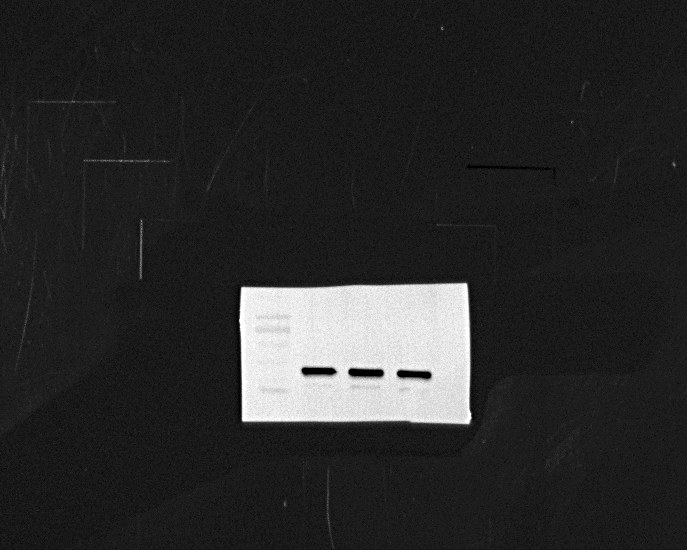

Supplement: Supplementary file 1 [file Data_Sheet_1.zip › original data1/Western blotting/WB-1/GAPDH-3.tif]

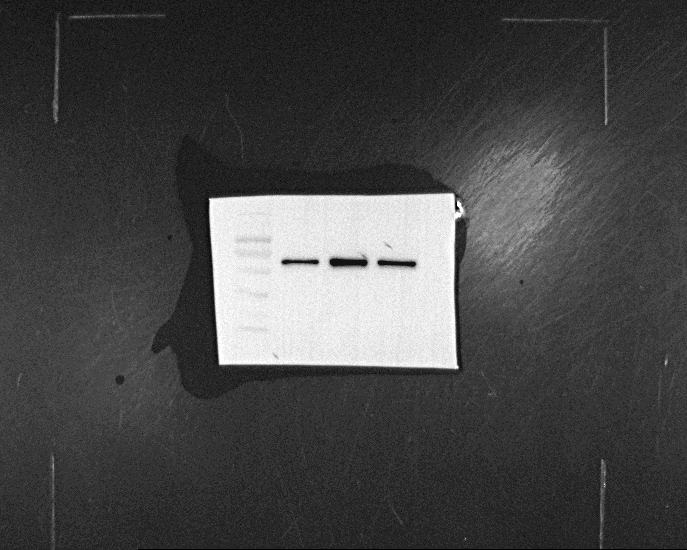

Supplement: Supplementary file 1 [file Data_Sheet_1.zip › original data1/Western blotting/WB-1/HDAC1-1.tif]

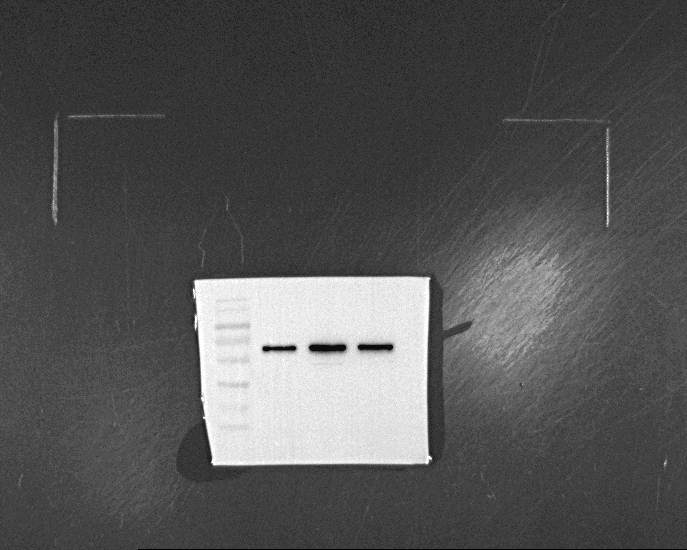

Supplement: Supplementary file 1 [file Data_Sheet_1.zip › original data1/Western blotting/WB-1/HDAC1-2.tif]

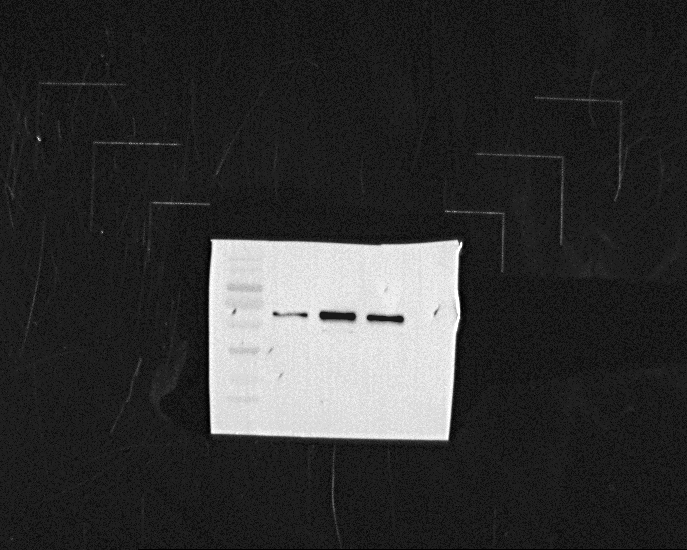

Supplement: Supplementary file 1 [file Data_Sheet_1.zip › original data1/Western blotting/WB-1/HDAC1-3.tif]

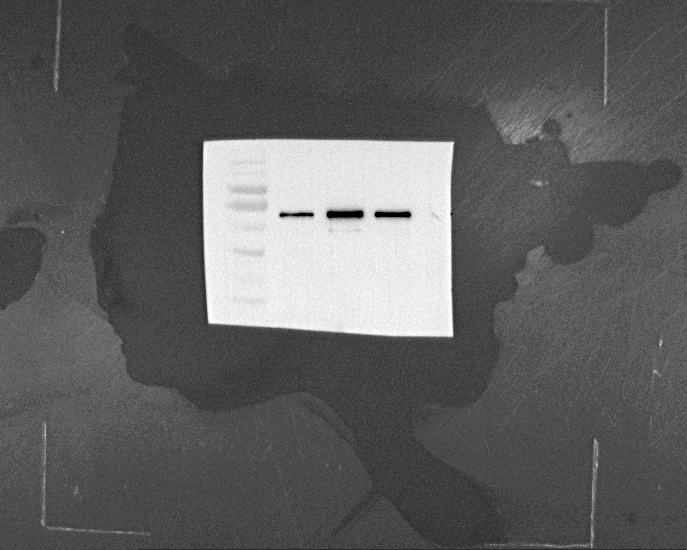

Supplement: Supplementary file 1 [file Data_Sheet_1.zip › original data1/Western blotting/WB-1/P-P65-1.tif]

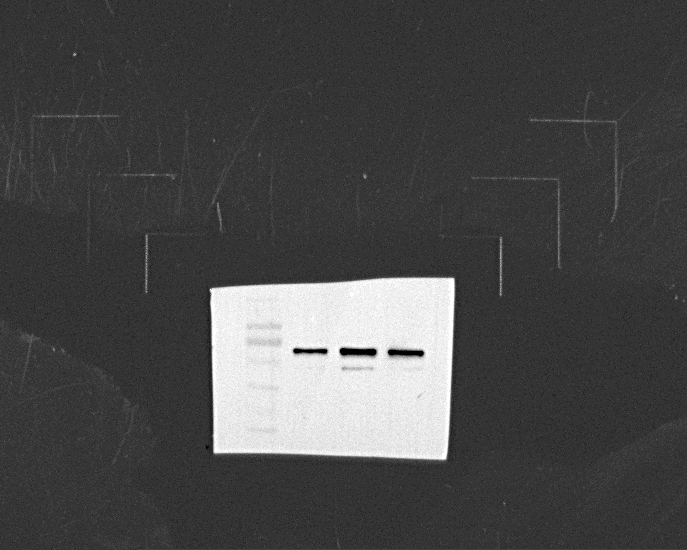

Supplement: Supplementary file 1 [file Data_Sheet_1.zip › original data1/Western blotting/WB-1/P-P65-2.tif]

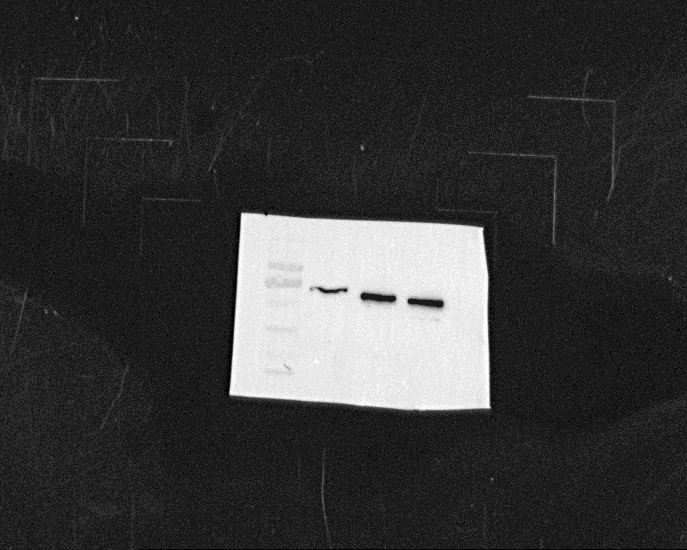

Supplement: Supplementary file 1 [file Data_Sheet_1.zip › original data1/Western blotting/WB-1/P-P65-3.tif]

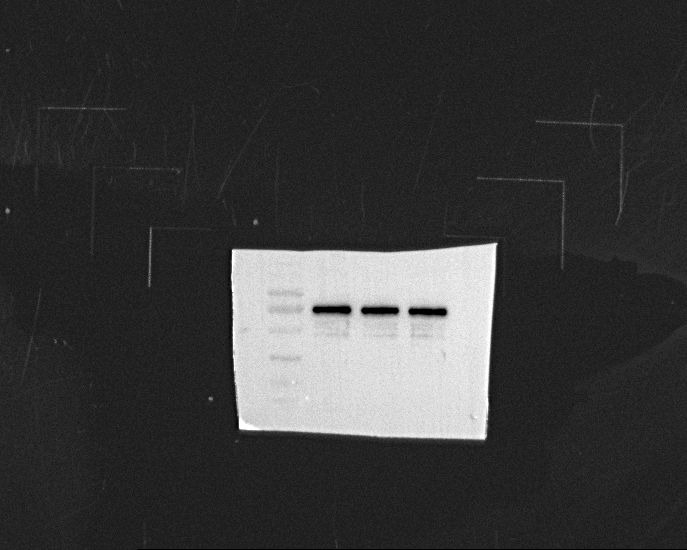

Supplement: Supplementary file 1 [file Data_Sheet_1.zip › original data1/Western blotting/WB-1/P65-1.tif]

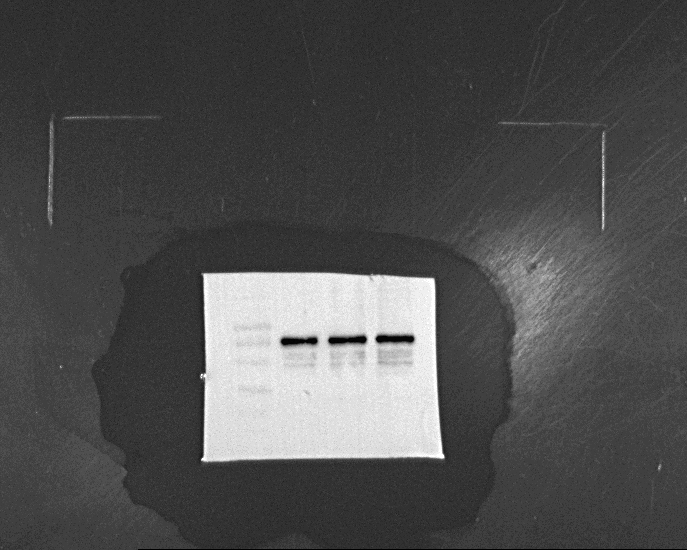

Supplement: Supplementary file 1 [file Data_Sheet_1.zip › original data1/Western blotting/WB-1/P65-2.tif]

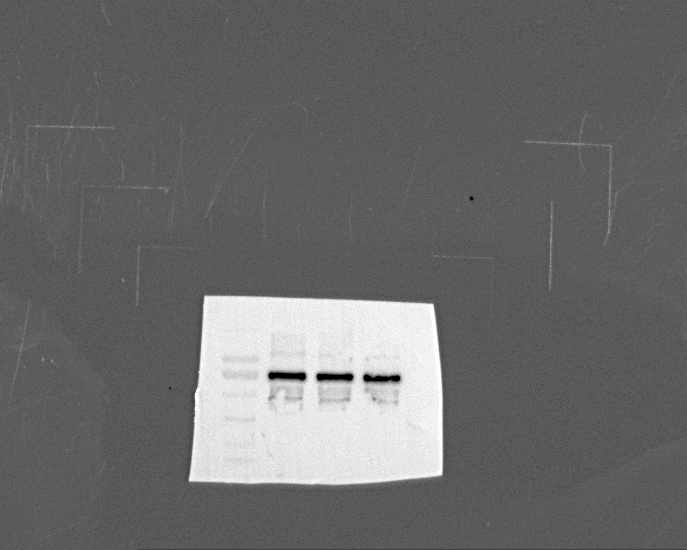

Supplement: Supplementary file 1 [file Data_Sheet_1.zip › original data1/Western blotting/WB-1/P65-3.tif]

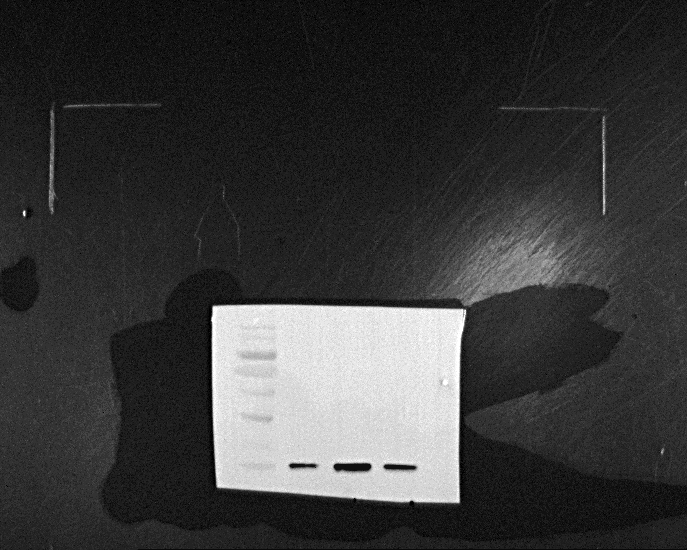

Supplement: Supplementary file 1 [file Data_Sheet_1.zip › original data1/Western blotting/WB-2/cith3-1.tif]

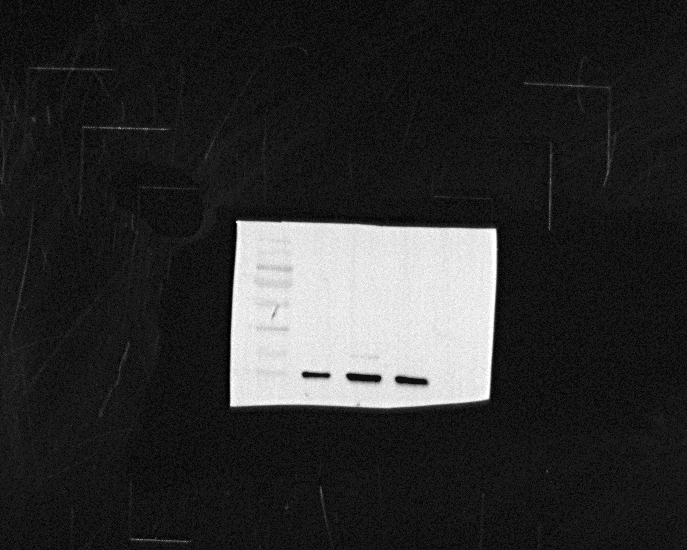

Supplement: Supplementary file 1 [file Data_Sheet_1.zip › original data1/Western blotting/WB-2/cith3-2.tif]

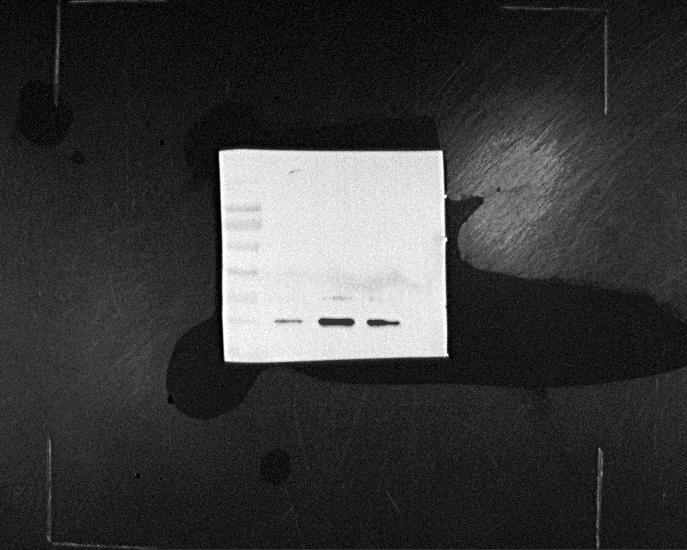

Supplement: Supplementary file 1 [file Data_Sheet_1.zip › original data1/Western blotting/WB-2/cith3-3.tif]

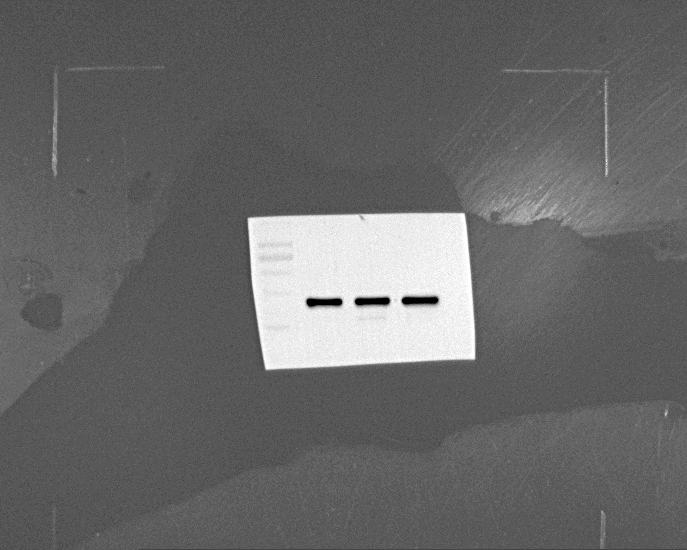

Supplement: Supplementary file 1 [file Data_Sheet_1.zip › original data1/Western blotting/WB-2/GAPDH-1.tif]

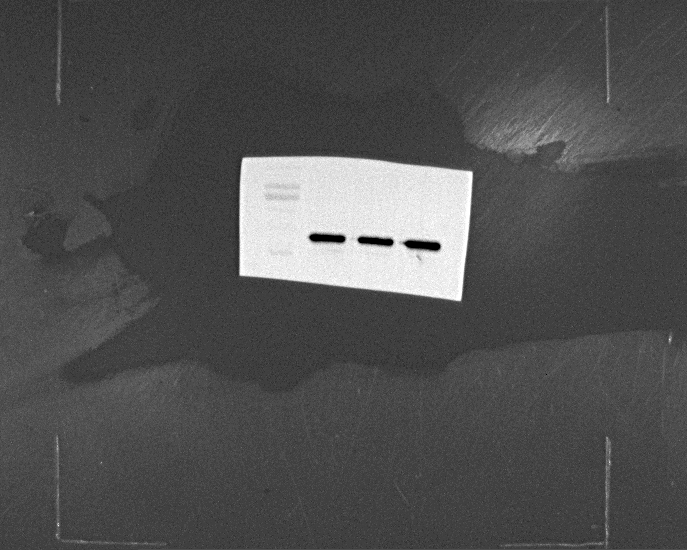

Supplement: Supplementary file 1 [file Data_Sheet_1.zip › original data1/Western blotting/WB-2/GAPDH-2.tif]

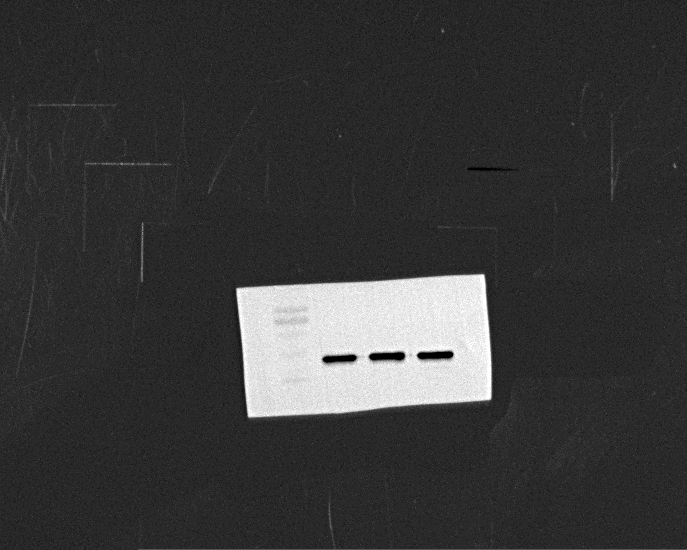

Supplement: Supplementary file 1 [file Data_Sheet_1.zip › original data1/Western blotting/WB-2/GAPDH-3.tif]

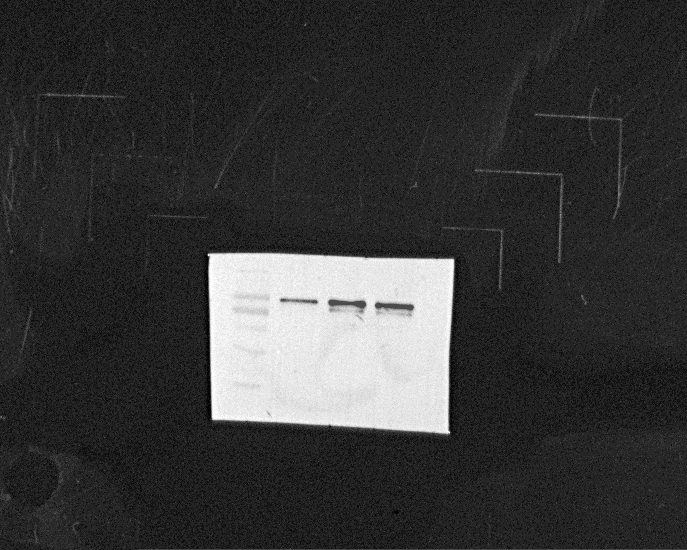

Supplement: Supplementary file 1 [file Data_Sheet_1.zip › original data1/Western blotting/WB-2/MPO-1.tif]

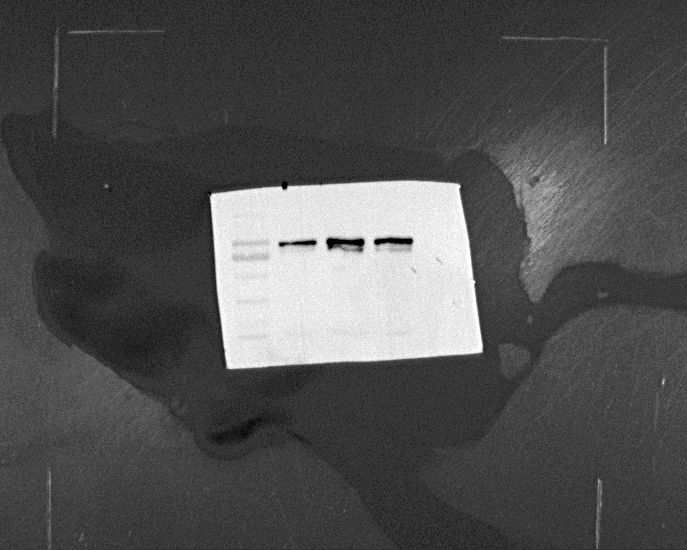

Supplement: Supplementary file 1 [file Data_Sheet_1.zip › original data1/Western blotting/WB-2/MPO-2.tif]

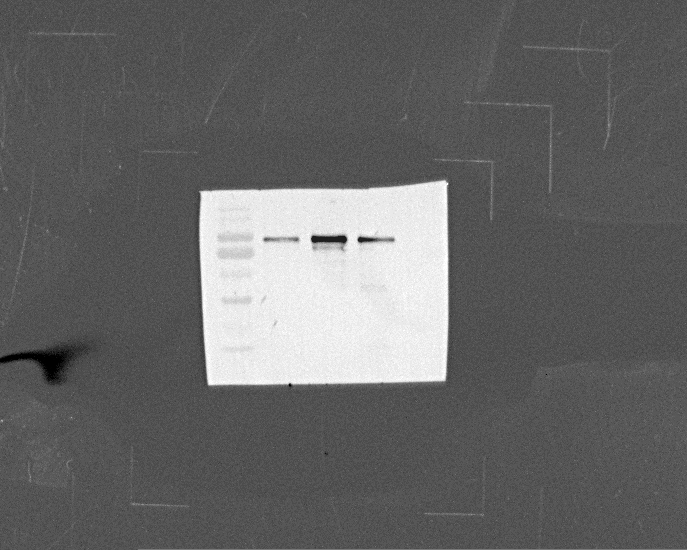

Supplement: Supplementary file 1 [file Data_Sheet_1.zip › original data1/Western blotting/WB-2/MPO-3.tif]

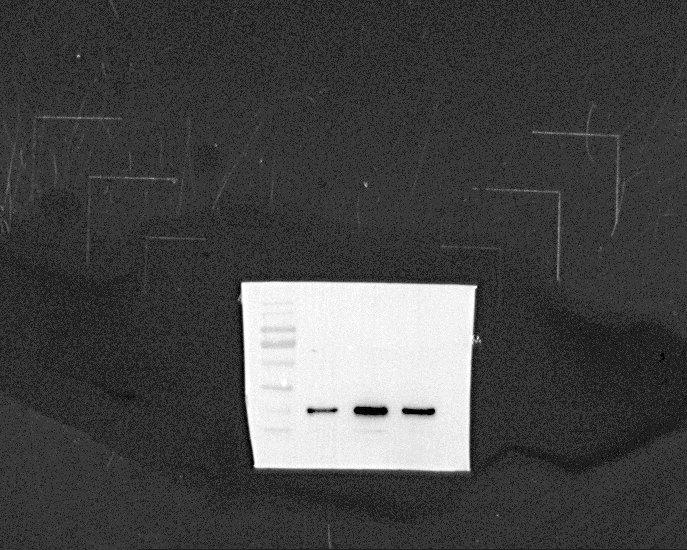

Supplement: Supplementary file 1 [file Data_Sheet_1.zip › original data1/Western blotting/WB-2/NE-1.tif]

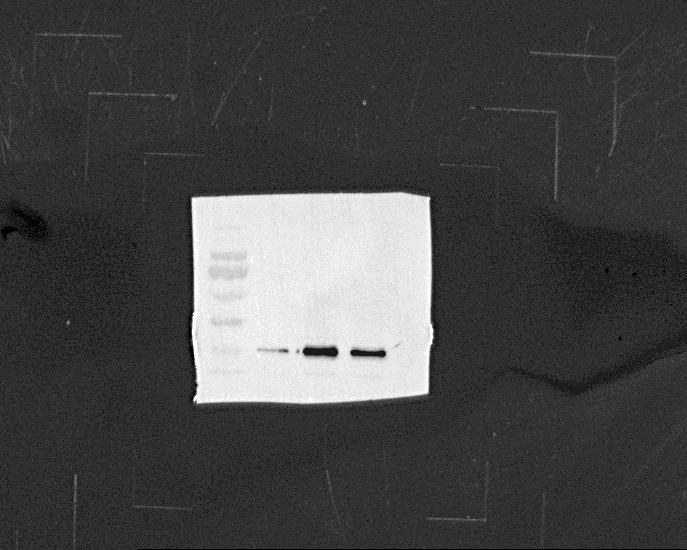

Supplement: Supplementary file 1 [file Data_Sheet_1.zip › original data1/Western blotting/WB-2/NE-2.tif]

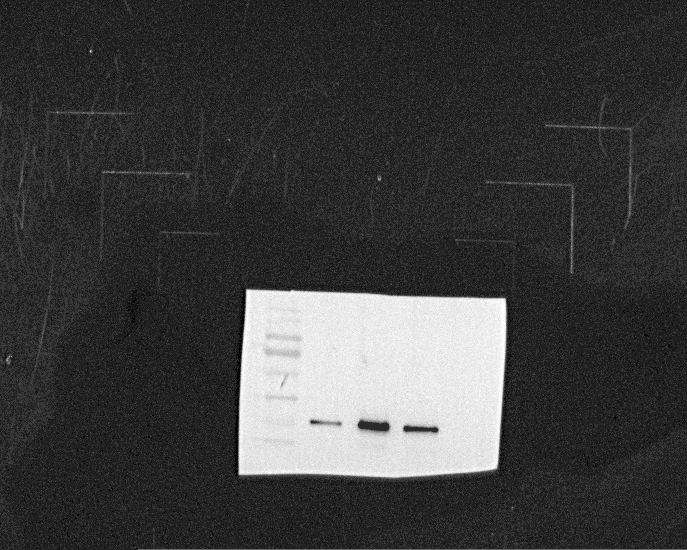

Supplement: Supplementary file 1 [file Data_Sheet_1.zip › original data1/Western blotting/WB-2/NE-3.tif]

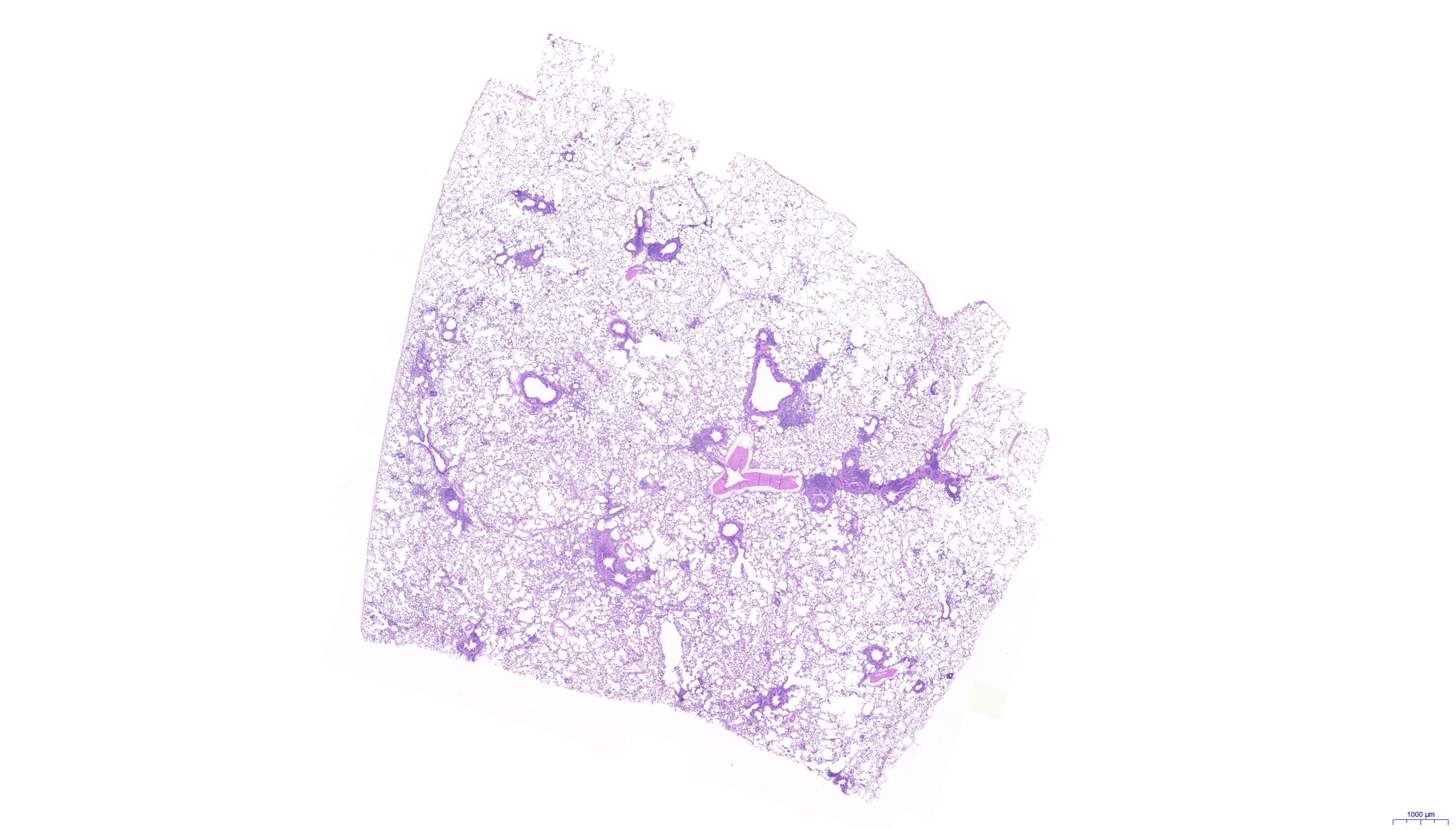

Supplement: Supplementary file 2 [file Data_Sheet_2.zip › original data3/HE/Con_1.0x (2).jpg]

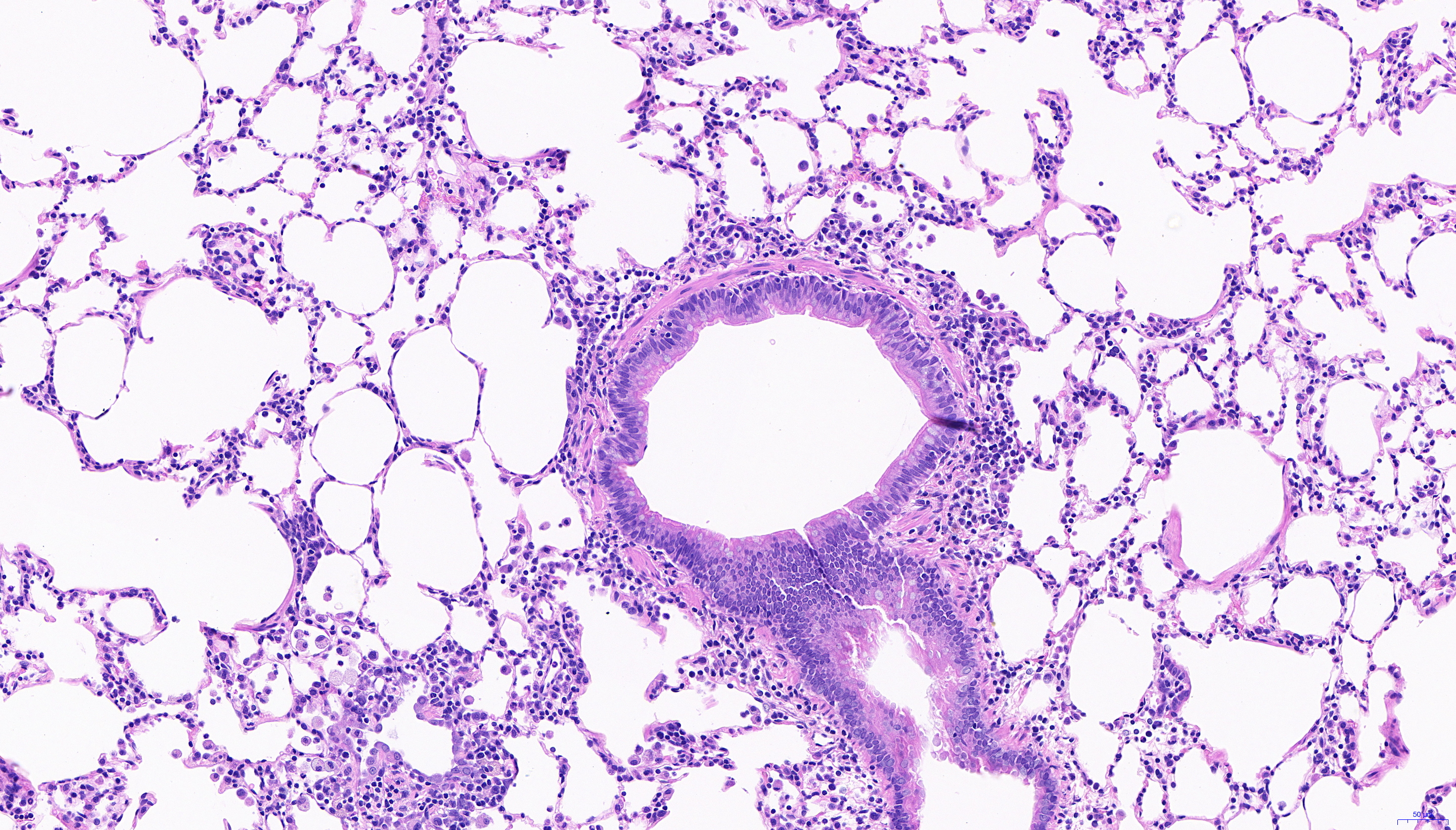

Supplement: Supplementary file 2 [file Data_Sheet_2.zip › original data3/HE/Con_20.0x.jpg]

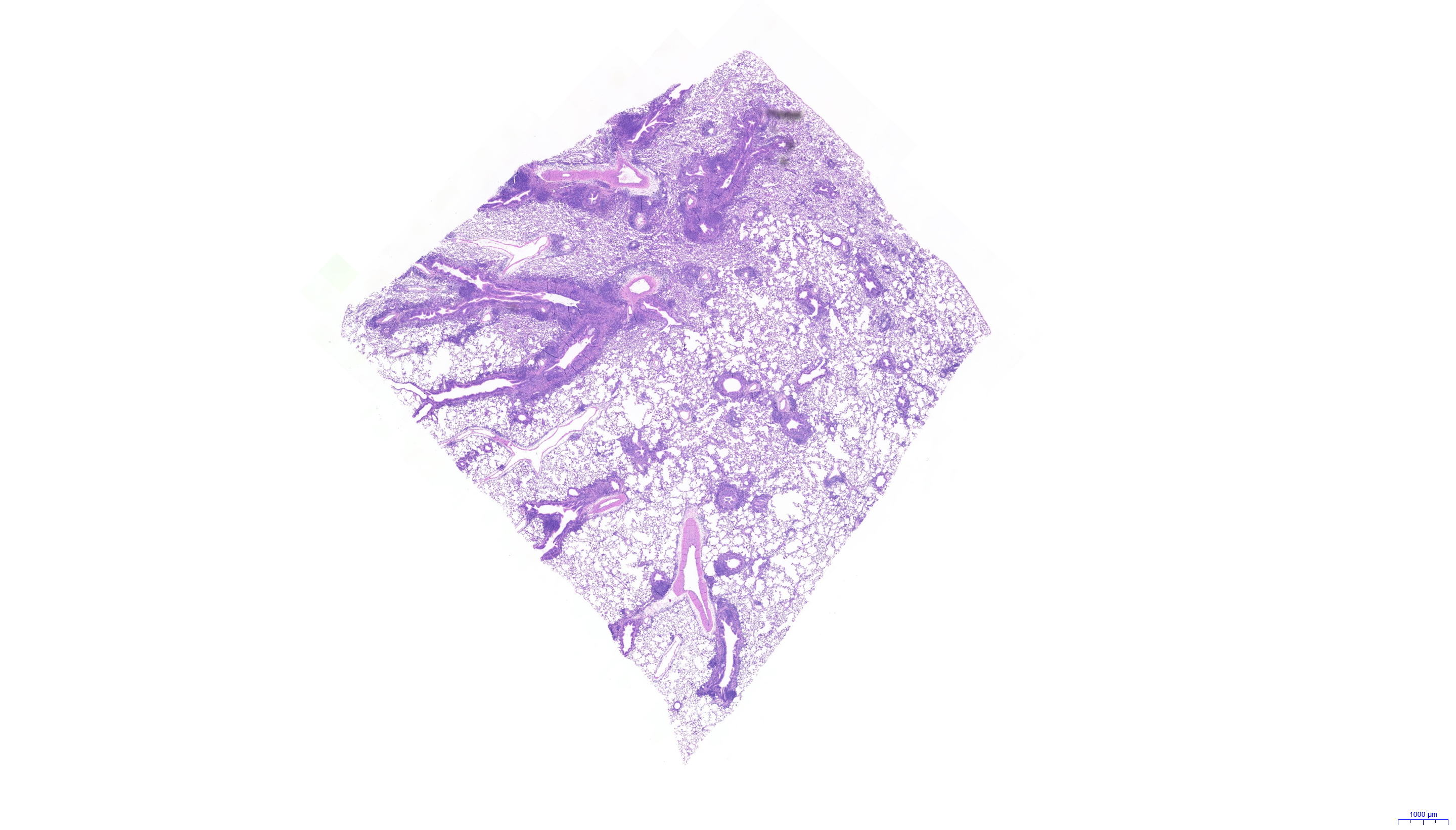

Supplement: Supplementary file 2 [file Data_Sheet_2.zip › original data3/HE/Mod_1.0x.jpg]

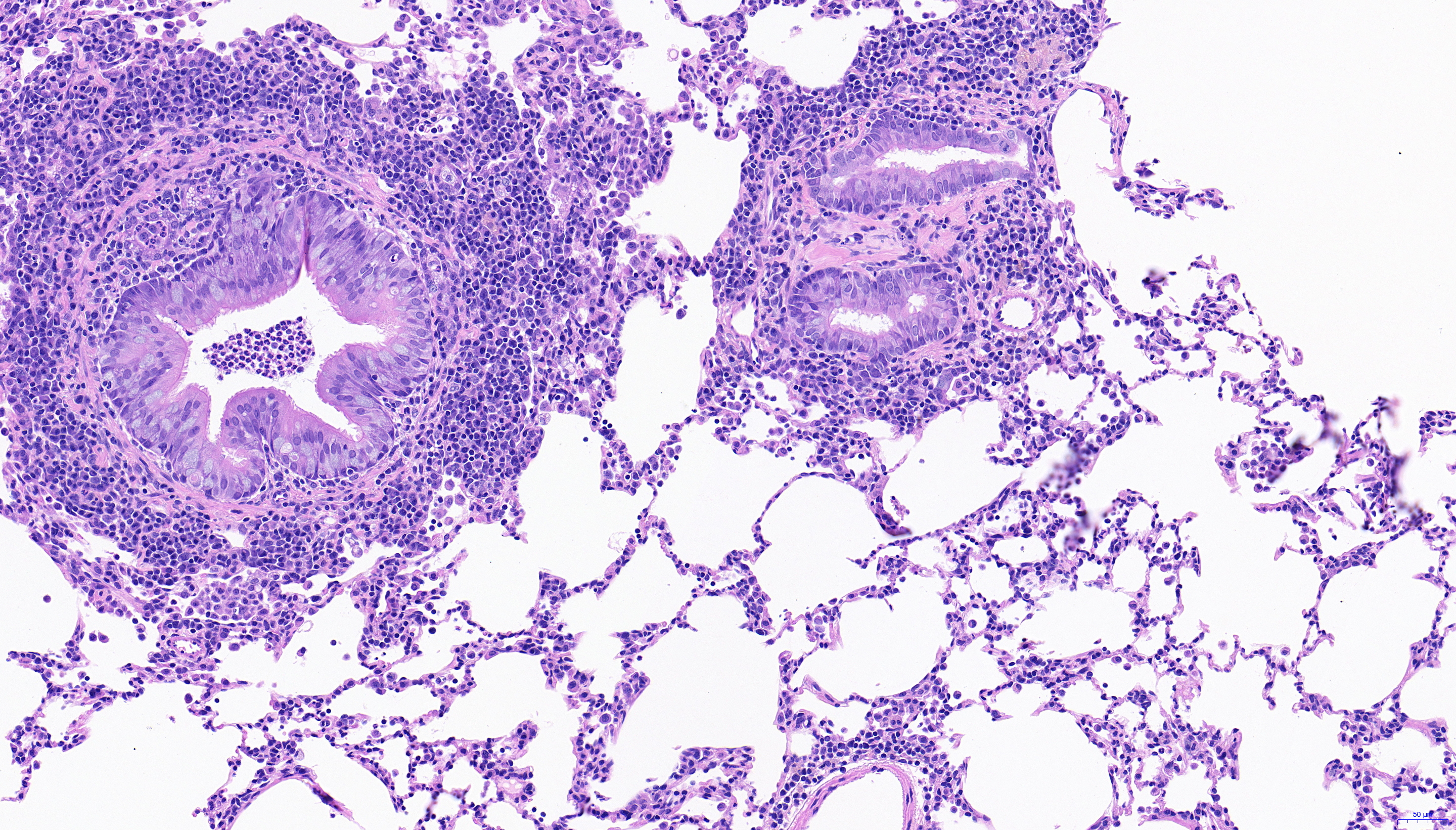

Supplement: Supplementary file 2 [file Data_Sheet_2.zip › original data3/HE/Mod_20.0x.jpg]

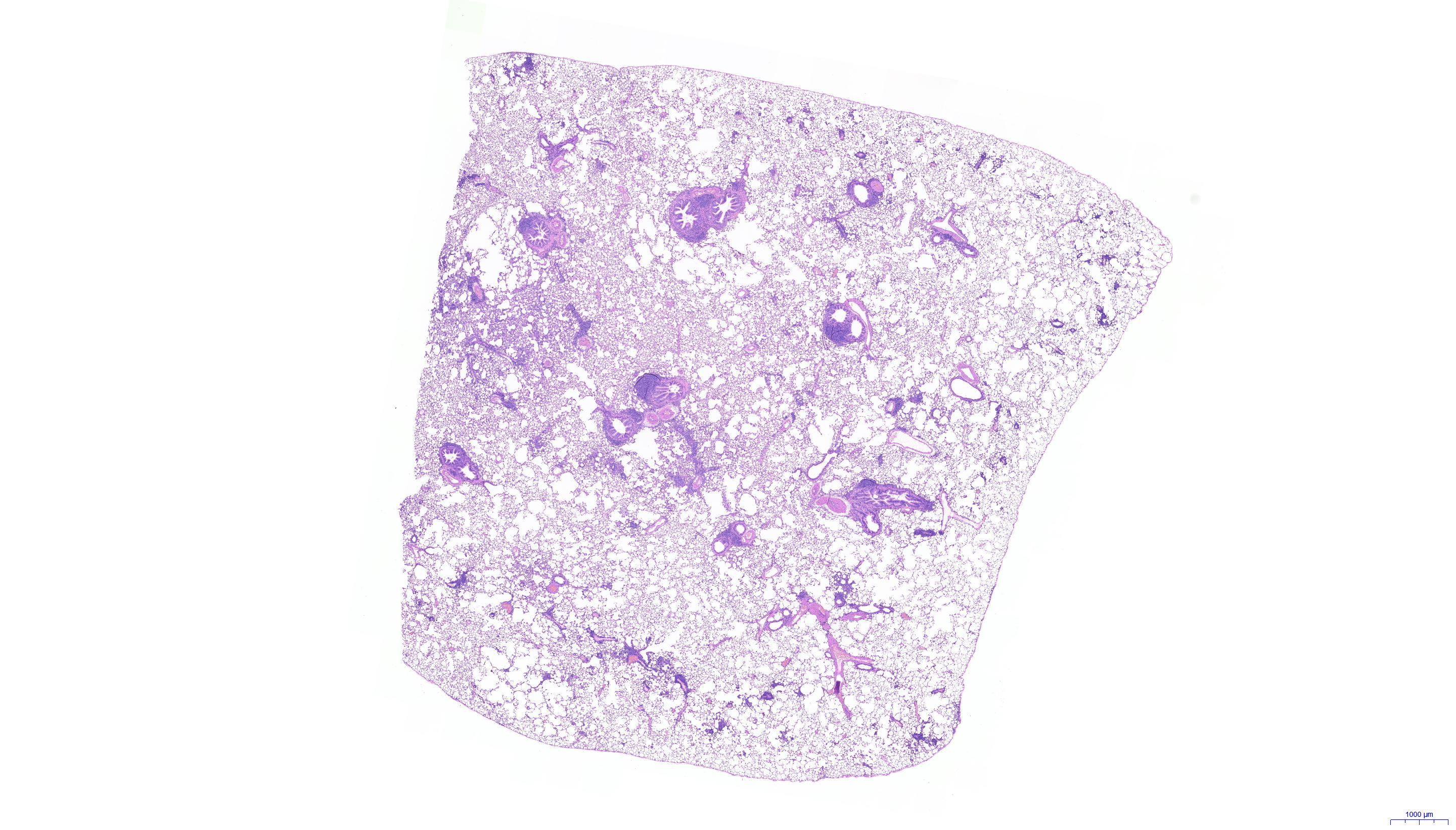

Supplement: Supplementary file 2 [file Data_Sheet_2.zip › original data3/HE/SQWF_1.0x.jpg]

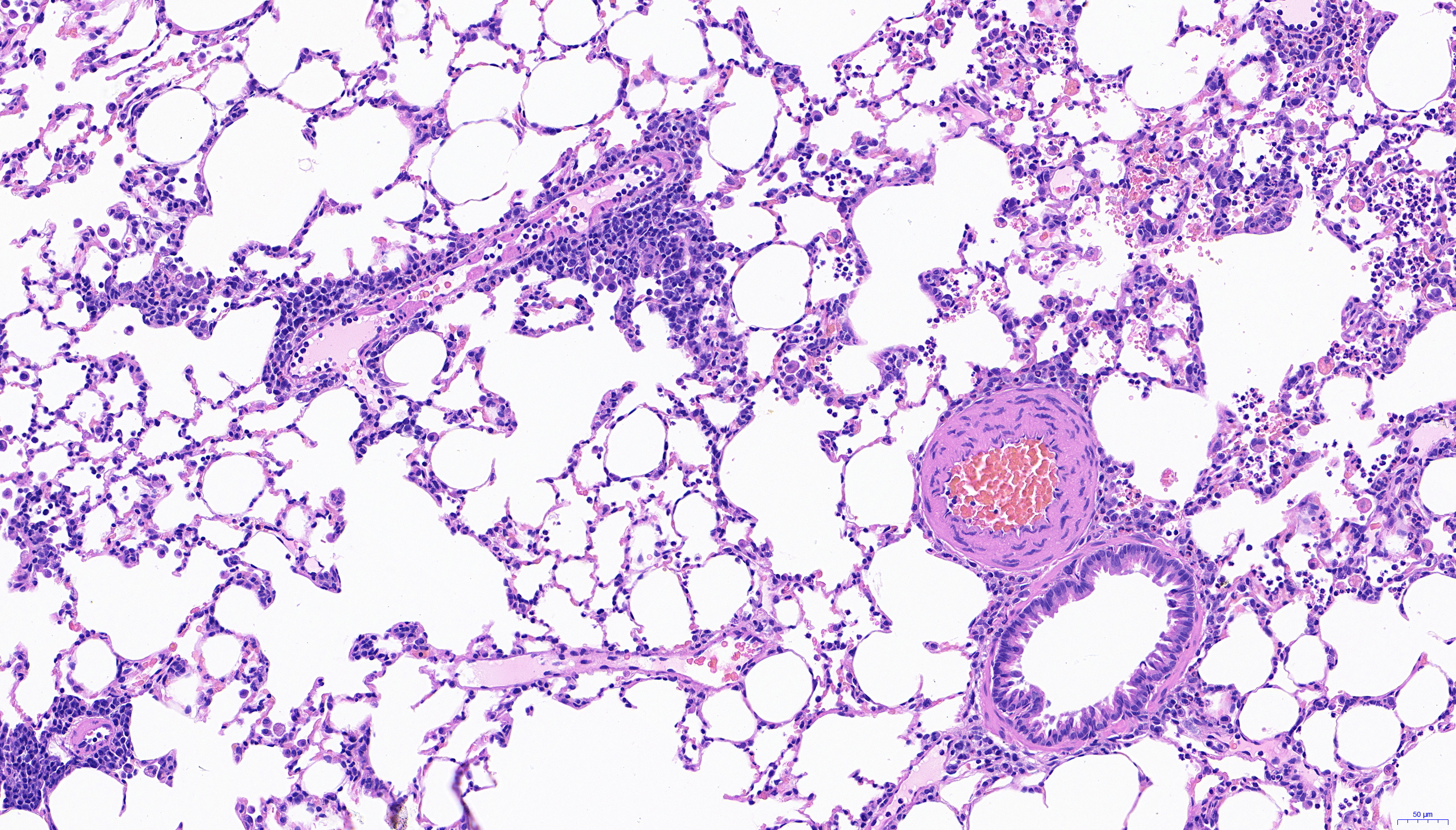

Supplement: Supplementary file 2 [file Data_Sheet_2.zip › original data3/HE/SQWF_20.0x.jpg]

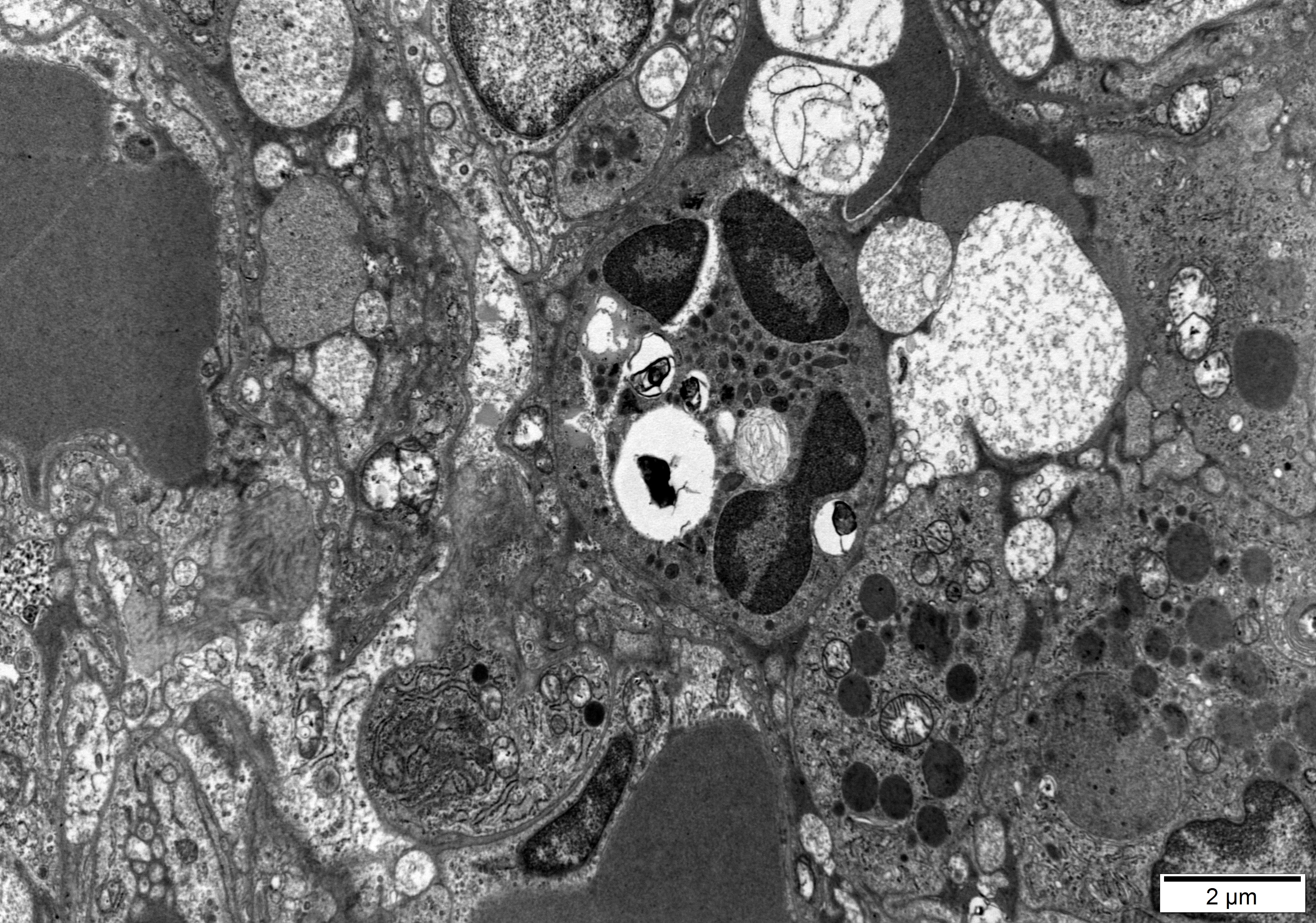

Supplement: Supplementary file 3 [file Data_Sheet_3.zip › (X10000)transmission electron microscope/Con_10000x_01.jpg]

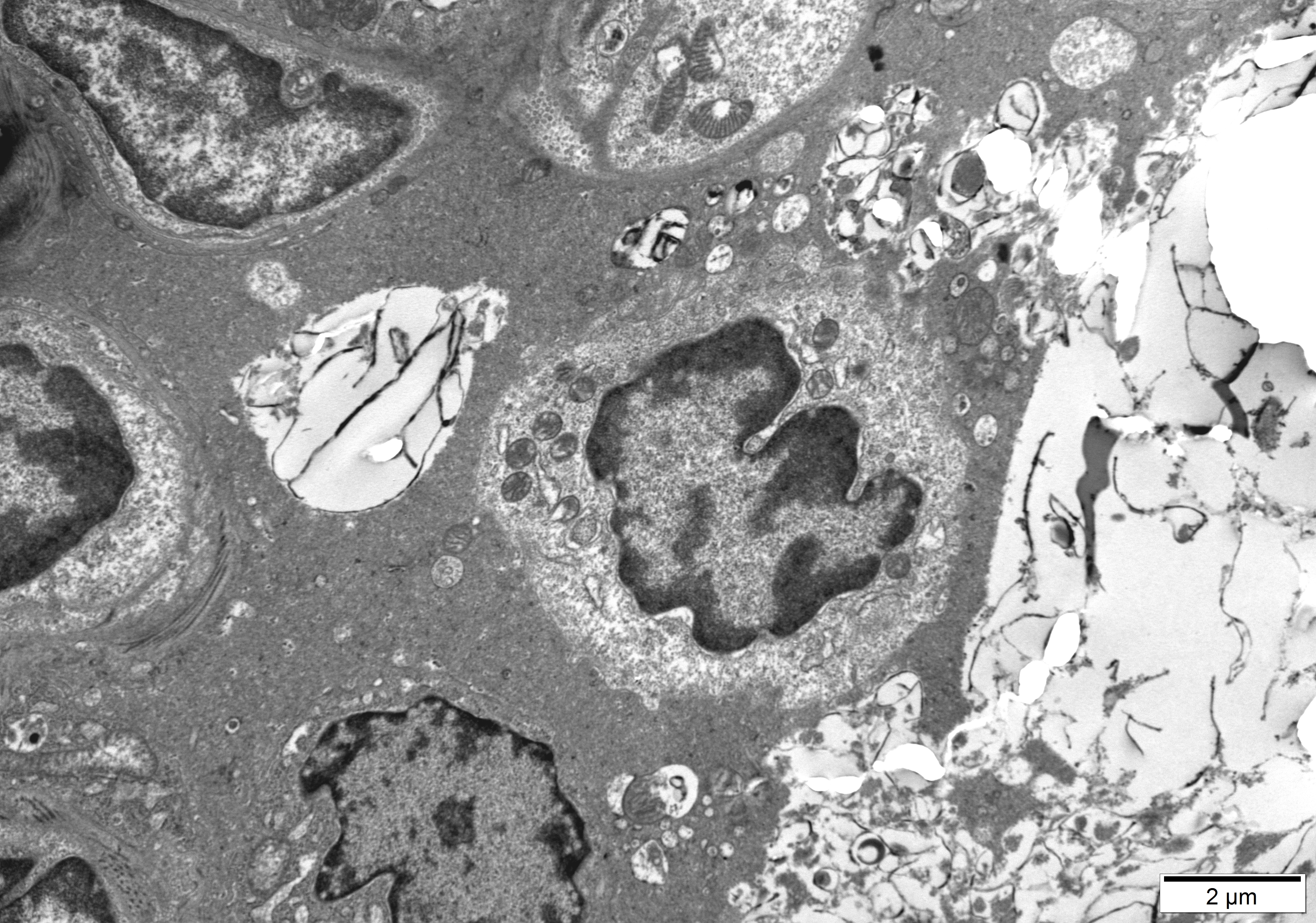

Supplement: Supplementary file 3 [file Data_Sheet_3.zip › (X10000)transmission electron microscope/Mod_10000x_05.jpg]

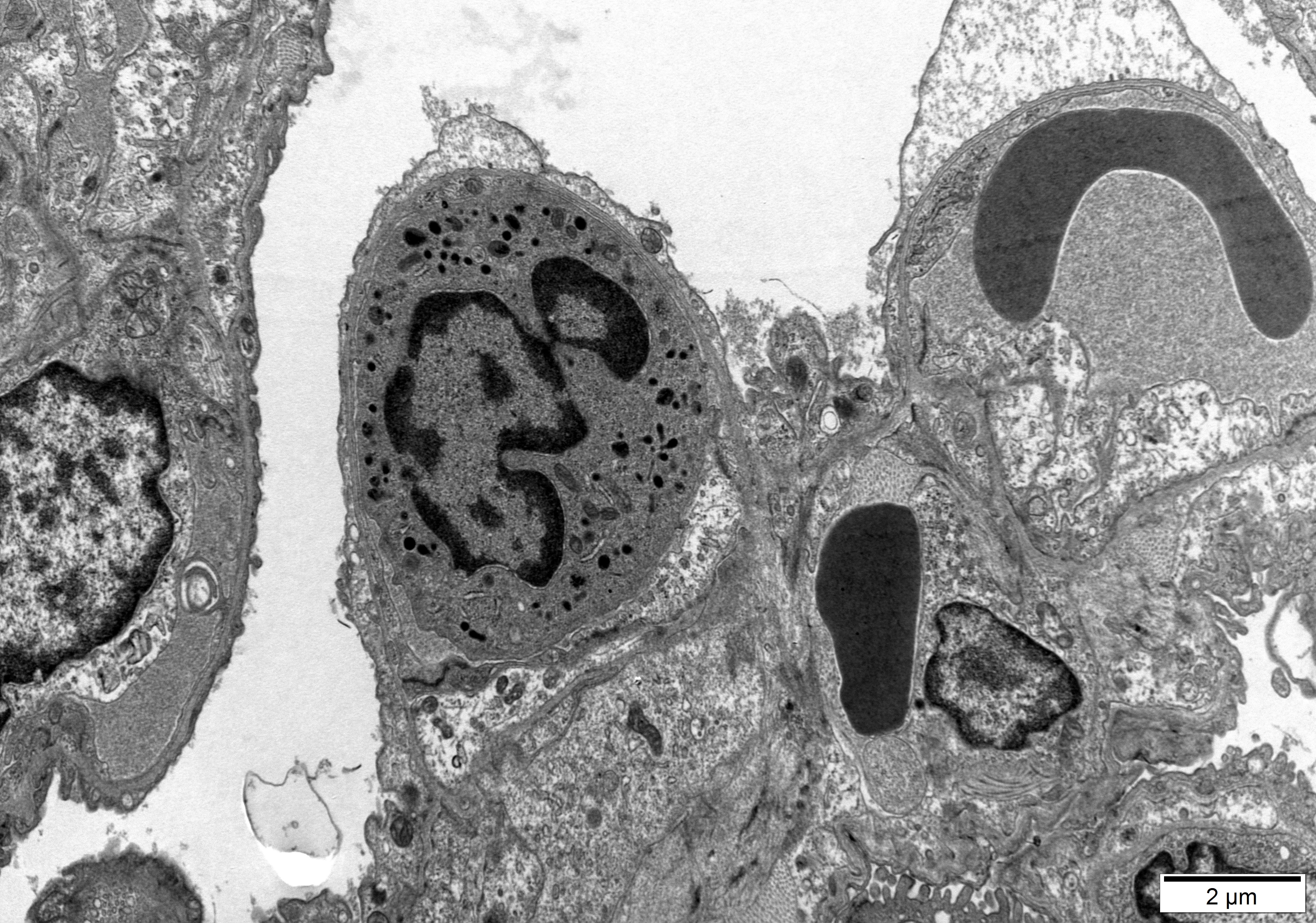

Supplement: Supplementary file 3 [file Data_Sheet_3.zip › (X10000)transmission electron microscope/SQWF-12_10000x_05.jpg]

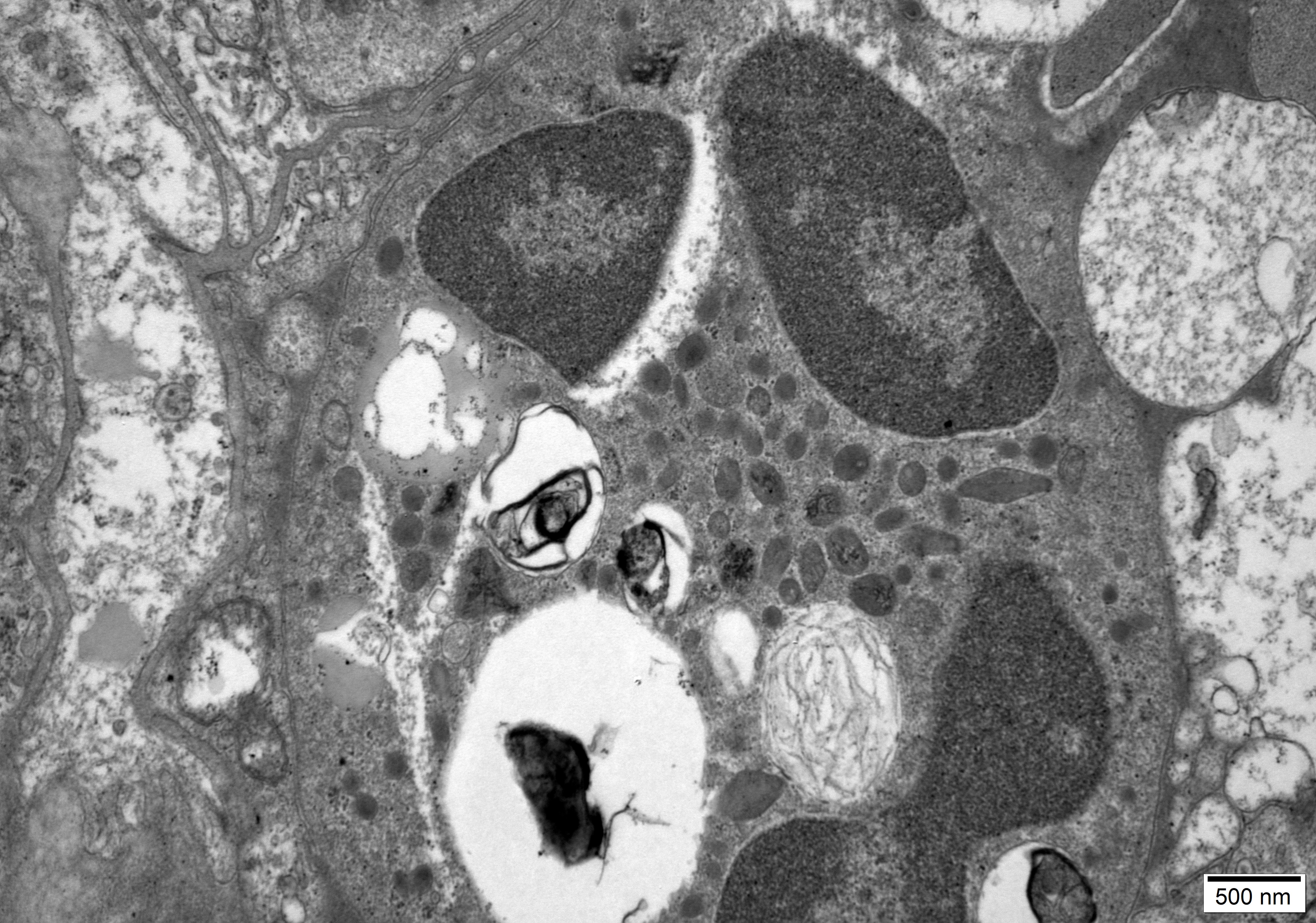

Supplement: Supplementary file 4 [file Data_Sheet_4.zip › (X25000)transmission electron microscope/Con_25000x_02.jpg]

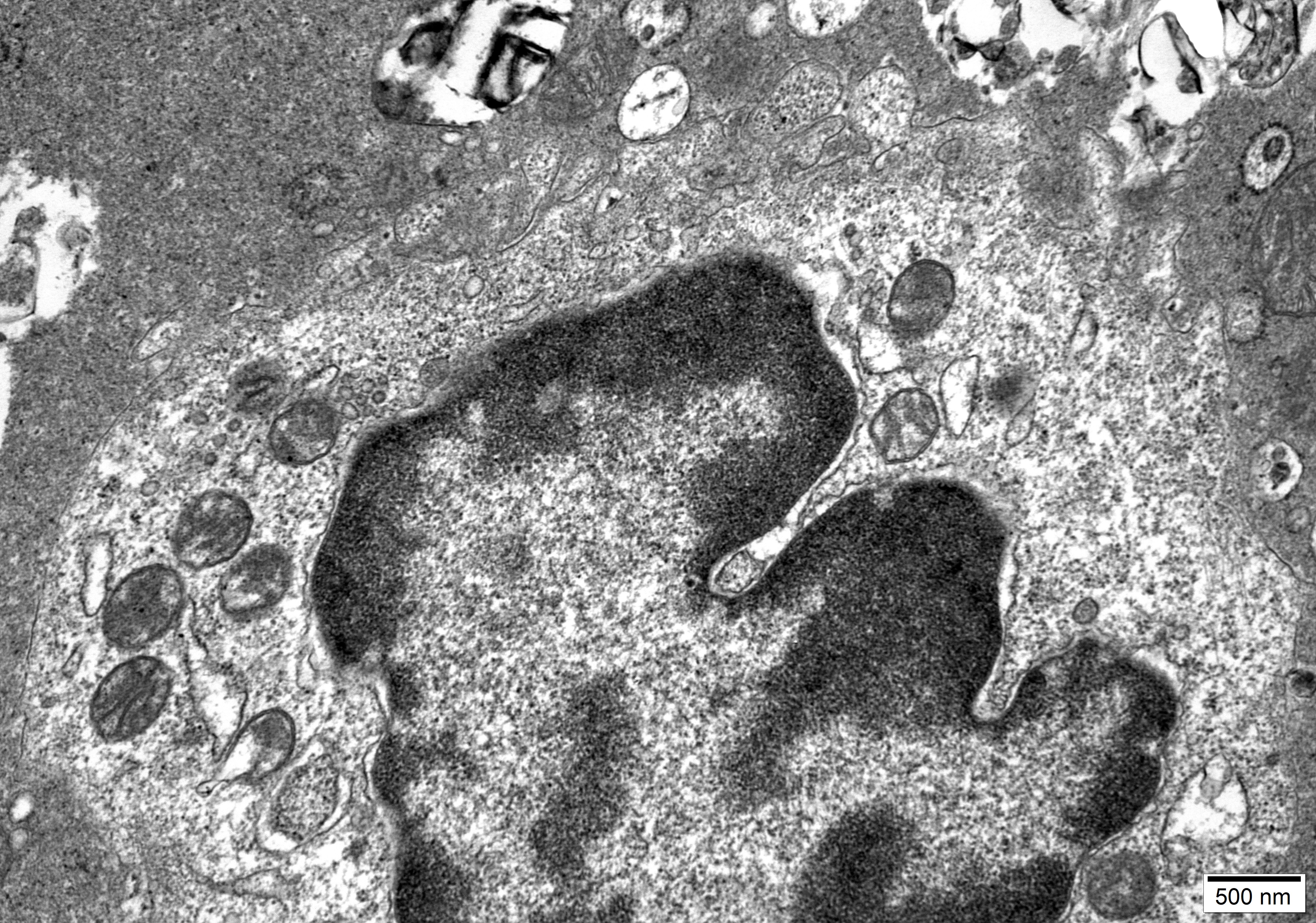

Supplement: Supplementary file 4 [file Data_Sheet_4.zip › (X25000)transmission electron microscope/Mod_25000x_06.jpg]

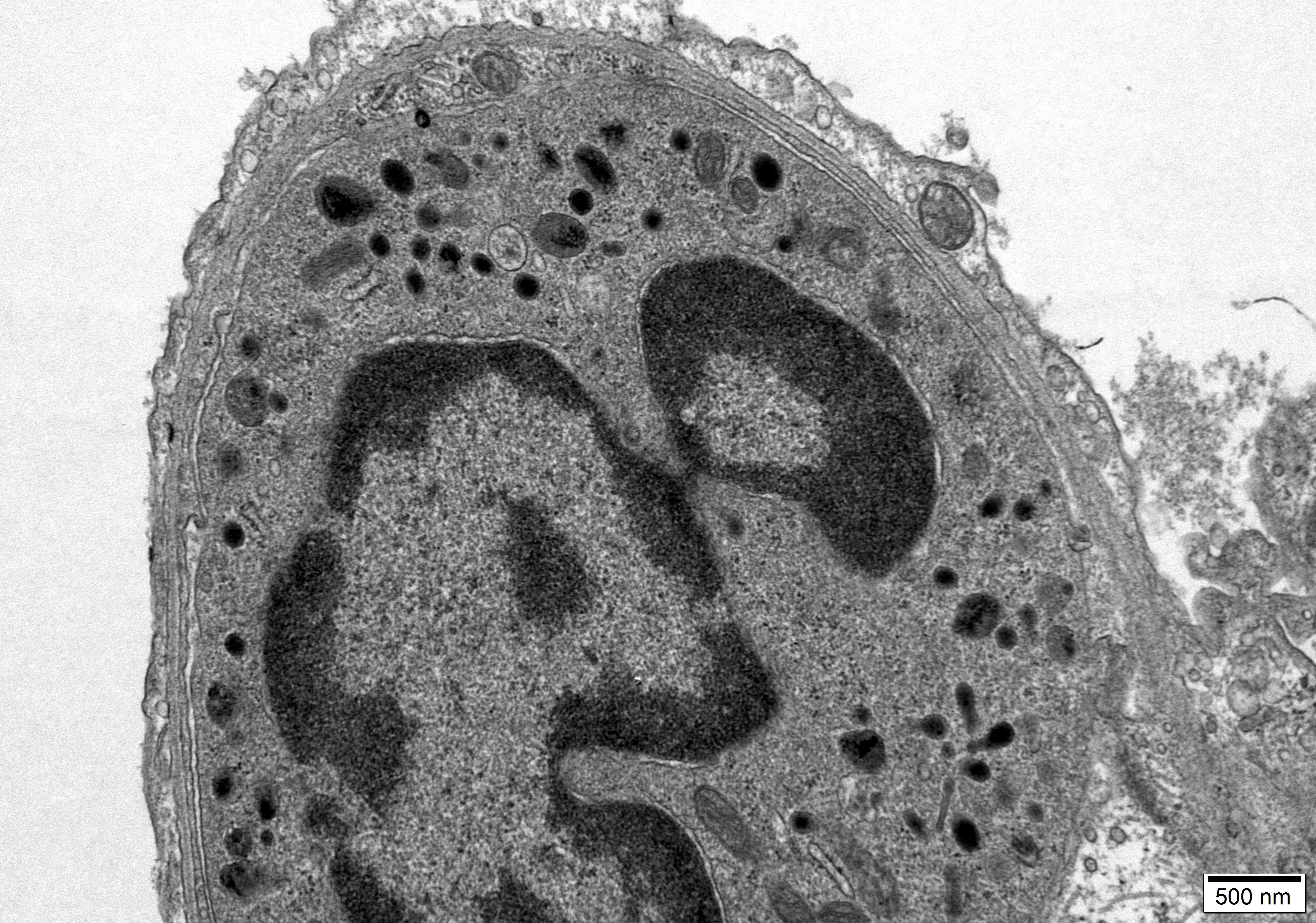

Supplement: Supplementary file 4 [file Data_Sheet_4.zip › (X25000)transmission electron microscope/SQWF-12_25000x_06.jpg]
